# Implementation effectiveness of the Long COVID and Fatiguing Illnesses Recovery Program (LC&FIRP): a multimodal Extension for Community Healthcare Outcomes (ECHO) model for clinician learning

**DOI:** 10.3389/frhs.2026.1912278

**Published:** 2026-10-23

**Authors:** Alexandra Zuber, Jonathan T. Rothwell, Job G. Godino, Jane C. Samaniego, Sydney P. Sharp, Douglas Taren, Elizabeth R. Unger, Jeanne Bertolli, Jin-Mann S. Lin, Joanna J. Regan, Amy J. Armistad, John D. Scott, Bruce B. Struminger, Christian B. Ramers

**Affiliations:** 1 Ata Health Strategies, LLC, Washington, DC, United States; 2 Laura Rodriguez Research Institute, Family Health Centers of San Diego, San Diego, CA, United States; 3 Herbert Wertheim School of Public Health and Longevity Science, UC San Diego, La Jolla, CA, United States; 4 Department of Pediatrics Nutrition Section, School of Medicine, University of Colorado, Aurora, CO, United States; 5 National Center for Emerging and Zoonotic Infectious Diseases, U.S. Centers for Disease Control and Prevention, Atlanta, GA, United States; 6 Project ECHO, University of New Mexico Health Sciences Center, Albuquerque, NM, United States; 7 Department of Medicine, University of Washington, Seattle, WA, United States; 8 School of Medicine, University of California San Diego, La Jolla, CA, United States; 9 School of Public Health, San Diego State University, San Diego, CA, United States

**Keywords:** community of practice, continuing medical education, ECHO, infection-associated chronic conditions, long COVID, implementation effectiveness

## Abstract

**Introduction:**

Long COVID and other infection-associated chronic conditions (IACC) present management challenges for primary care clinicians and often require patient access to multidisciplinary specialists. To strengthen primary care capacity, the Long COVID and Fatiguing Illnesses Recovery Program (LC&FIRP) implemented a multi-modal learning model combining Extension for Community Healthcare Outcomes (ECHO) case-based learning with complementary educational modalities. The ECHO learning component was evaluated for effectiveness in a Type II hybrid design randomized control trial, and an implementation evaluation assessed implementation of the full LC&FIRP model to inform future implementation and scale-up.

**Methods:**

We conducted a qualitatively driven mixed-methods implementation evaluation of the LC&FIRP model, assessing acceptability, feasibility, fidelity, sustainability, and scalability. Participants comprised clinicians from a federally qualified health center serving predominantly uninsured, low-income, and medically underserved patients in San Diego, CA. All trial clinicians were offered monthly webinars and quarterly asynchronous online short courses; intervention clinicians were additionally offered weekly ECHO sessions. Data sources were session surveys at the end of every session or short-course, quarterly clinician follow-up surveys, and interviews with participants, subject matter experts (SMEs) and clinic administrators at program end.

**Results:**

Acceptability findings reflected high clinician satisfaction and perceived utility of the weekly and monthly offerings but moderate sustained engagement and negligible participation in the quarterly short courses. Clinicians valued most the interactive peer exchange; practical diagnostic and treatment approaches; and lessons from SMEs with lived experience. Feasibility barriers included competing clinical and administrative demands and the time commitment required for participation. Most program components were implemented with fidelity but fewer weekly ECHO sessions and case presentations were delivered than planned. Sustainability and scalability showed early promise with the continuation of weekly and monthly offerings after trial end, but future barriers remain.

**Discussion:**

This study demonstrates that a multi-modal program combining ECHO case-based learning with complementary educational offerings may be feasibly implemented and is acceptable to clinicians for supporting primary care management of complex chronic conditions, although sustaining engagement and scaling the model will require adaptations to reduce participation burden and resource requirements.

## 1 Introduction

Before the Extension for Community Healthcare Outcomes (ECHO) model became an evidence-based intervention for clinical and public health workforce development, it was established to empower primary care clinicians to deliver specialty Hepatitis C care in underserved settings in New Mexico (1). Using a hub-and-spoke design, the ECHO connects a multi-disciplinary team of specialists with a network of frontline health clinicians at different clinic sites using weekly, virtual, case-based learning sessions (2). Over time, this model showed effectiveness in the treatment of other complex chronic health issues such as opioid use disorder and hospice care (3–6), and in addressing underlying barriers and expanding access to specialty care, particularly in areas where clinical treatment approaches were newly emerging and lack clear clinical guidelines (7, 8).

These results make the model particularly suited for addressing the challenges in the management of a newly emergent condition- Long COVID. Long COVID is a debilitating infection-associated chronic condition (IACC) that occurs after SARS-CoV-2 infection and is present for at least 3 months as a continuous, relapsing and remitting, or progressive disease state that affects one or more organ systems (9–12). Several Long COVID symptoms also occur in patients with myalgic encephalomyelitis/chronic fatigue syndrome (ME/CFS), including fatigue, post-exertional malaise, and cognitive impairment, among others, and ME/CFS itself can be a presentation of Long COVID (13–15). Another shared feature of these conditions is the need for patients to consult multiple specialties, which places special demands on patient time and resources (16, 17). In contexts serving people from low-income and racially and ethnically diverse populations, such as federally qualified health centers (FQHCs), patients may face additional barriers to access to specialty care, resulting in treatment delays (18). The ECHO model, with its emphasis on virtual learning sessions connecting primary care clinicians with multi-disciplinary experts, is a promising approach to improving uptake of multidisciplinary specialist clinical knowledge among primary care providers without requiring specialist referrals for patients with Long COVID, ME/CFS and potentially other IACC.

A large volume of studies have documented the effectiveness of the ECHO model (2–4, 6, 19–21), however, only three randomized controlled trials (RCTs) were identified (22–24). Given the wide dissemination of the ECHO model (8, 25), it is critical that a prospective, randomized evidence base be expanded to soundly support further implementation (26). From December, 2021 to October, 2024, the U.S. Centers for Disease Control and Prevention (CDC) supported Family Health Centers of San Diego (FHCSD) and other partners to conduct a randomized controlled trial of the ECHO learning model for the management of Long COVID, ME/CFS and other IACC (27). This study and its primary outcomes are described in two unpublished companion papers, referred hereafter as Sharp, S. et al. (48) and Zuber, A. et al. (49) focused on the “Long COVID and Fatiguing Illnesses Recovery Program (LC&FIRP).”

In recent years, researchers have raised concerns about the slow, sequential progression from clinical efficacy to effectiveness to implementation research, which can delay the translation of evidence into routine practice (28, 29). Effectiveness-implementation hybrid study designs can address this gap by assessing implementation outcomes concurrently with primary patient or clinician outcomes (28–30) as distinct research questions. LC&FIRP was prospectively designed as a Type II effectiveness-implementation hybrid trial. The co-primary research aims were to assess the incremental effectiveness of ECHO case-based learning over other educational modalities on: (1) patient and (2) clinician outcomes (28, 29). The secondary aim, reported in this paper, was to assess implementation effectiveness of the full LC&FIRP educational model- including weekly case-based ECHO sessions, monthly webinars, and quarterly asynchronous online short courses- by examining acceptability, feasibility, fidelity, sustainability and scalability. The implementation evaluation was intended to identify strengths, barriers, and potential adaptations to inform future delivery and scale-up of the model.

## 2 Methods

### 2.1 LC&FIRP program model

The clinical objective of LC&FIRP was to improve primary care management of Long COVID, ME/CFS, and other IACC through the uptake and application of multidisciplinary clinical knowledge and recommendations. The ECHO learning model—particularly the weekly case-based virtual community of practice—served as the principal implementation strategy for translating multidisciplinary expertise into primary care practice. Monthly webinars and quarterly asynchronous short courses—offered to control and intervention clinicians alike- provided additional mechanisms for dissemination (27). We define our hybrid study design as Type II because it simultaneously evaluated the effectiveness of the clinical intervention on clinician and patient outcomes and the implementation of the strategies intended to support its uptake in primary care practice (28, 29).

In Type II hybrid designs, the distinction between a clinical intervention and an implementation strategy can be unclear, and some interventions may function as implementation strategies or as clinical interventions in their own right depending on how they are used (29, 31, 32). In LC&FIRP, ECHO was operationalized as the principal implementation strategy for translating multidisciplinary clinical knowledge and recommendations into primary care practice, within a broader strategy bundle that included webinars and asynchronous short courses as complementary dissemination mechanisms.

LC&FIRP primarily focused on Long COVID and ME/CFS, while also addressing other post-infectious illnesses and commonly associated conditions and manifestations, including POTS/dysautonomia, post-exertional malaise, and mast cell activation syndrome. The model comprised three virtual, educational components: 1) weekly ECHO case-based virtual community of practice sessions (hereafter referred to as “weekly ECHO sessions”) provided to a small group of trial clinicians in the intervention group (*n* = 10); 2) monthly webinars that were open to all trial clinicians in both intervention and control groups (*n* = 20) and a national audience of primary care clinicians; 3) quarterly asynchronous online short-courses (hereafter referred to as “quarterly short-courses”) open to all trial clinicians and national primary care clinicians. Webinars and quarterly short courses were made available to a national audience to help disseminate lessons learned to a wider audience in a timely way. The three modalities are detailed below by clinician trial condition.

LC&FIRP drew upon a national pool of multi-disciplinary subject matter experts (SMEs) from academic medical centers, who participated in both session types as presenters and panelists. In addition, the program included as SMEs “lived experience experts”- or people living with Long COVID, ME/CFS, or other IACC illnesses who could share their unique knowledge and experience from the patient perspective. A WhatsApp group chat provided a forum for discussion and resource-sharing between SMEs participating in weekly ECHO sessions and monthly webinars. Across both formats, participant and SME feedback, together with emerging scientific literature, informed the selection of topics for subsequent sessions, allowing the curriculum to evolve alongside the rapidly developing evidence base. A complete list of topics for weekly ECHO session, monthly webinar, and quarterly asynchronous short courses is provided in Supplementary materials.

#### 2.1.1 Control condition

All trial clinicians (*n* = 20) were invited to participate in the control condition of monthly webinars and quarterly short courses. Monthly webinars were 60 min in length and held over Zoom videoconference. They comprised a 30-minute didactic presentation by one or more SMEs on a patient care topic in Long COVID, ME/CFS or other IACC, and an interactive component of questions and answers between the audience and the SME(s). The monthly webinar series began with a broad overview of both conditions before progressing to more in-depth exploration of Long COVID manifestations, emerging evidence, and approaches to clinical management.

Nine quarterly short courses were also offered during the trial period. Each course was designed to take less than one hour to complete and was offered on the University of Colorado's web page. The first four quarterly short courses were developed by consolidating key didactic content from the weekly ECHO sessions into module-based courses. For Courses 5–9, the LC&FIRP study team adopted a single-topic format, allowing each course to explore one subject in greater depth rather than combining multiple didactic presentations from the previous quarter.

#### 2.1.2 Intervention condition

In addition to these two educational modalities, intervention clinicians also were invited to attend weekly ECHO sessions. In weekly ECHO sessions, clinicians received didactic presentations on patient care topics by one or more SMEs on a multidisciplinary SME panel, such as on novel diagnostic practices, treatment approaches, and advents in research/ science. The sessions were 60 min in length; they started with a 15-minute SME didactic presentation, followed by 10-minutes of interactive question and answer period where clinicians could ask questions of the presenters. The majority of the session then focused on an interactive case presentation and discussion, where a participating clinician presented a real patient case and received individualized diagnostic and therapeutic recommendations from the SME panel. If no case was scheduled, the didactic presentation and interactive question and answer period extended to fill the 60 min.

The weekly ECHO session series began with an overview of the LC&FIRP program and participant expectations, followed by introductory sessions addressing the definitions and diagnostic criteria for Long COVID and ME/CFS. Subsequent weekly ECHO sessions focused more extensively on the clinical management of these complex conditions, including symptom-specific management and treatment approaches, practical strategies for primary care, and multidisciplinary considerations.

### 2.2 Implementation evaluation design

The implementation effectiveness study utilized mixed qualitative and quantitative methods to assess implementation outcomes. Because clinicians in both trial groups received components of the LC&FIRP implementation-strategy bundle, the implementation evaluation assessed outcomes among both groups. Findings are distinguished between educational modality.

Quantitative data soliciting perspectives of trial clinicians on Likert scales were collected in electronic surveys on a quarterly and individual session basis. Clinician participation was documented through Zoom records. Qualitative perspectives of trial clinicians, SMEs, and clinic administrators were collected through semi-structured interviews. Participating clinicians and SMEs were selected as study subjects due to their direct involvement in the program, and clinic administrators were selected for their perspectives on the acceptability and feasibility of the model in their clinic settings.

All study instruments were developed collaboratively with the LC&FIRP research team and interview guides were iteratively revised through the interview process (all instruments are available in the Supplementary materials). Researcher characteristics and reflexivity statement are also in the Supplementary materials. The study was registered at Clinicaltrials.gov (NCT05167227) and was reviewed and approved by the Institutional Review Board of San Diego State University, approval number HS-2021-0241. This study was funded by the U.S. CDC and was conducted by a consortium of practice-based and academic institutions that include FHCSD, Ata Health Strategies, ECHO Institute at the University of New Mexico Health Sciences Center, University of Colorado School of Medicine, and University of Washington School of Medicine. This study was reported in accordance with the Standards for Reporting Qualitative Research (SRQR), given its emphasis on qualitative methods (33). The SRQR checklist of this study is available in the appendix.

### 2.3 Setting and participants

The study was conducted at Family Health Centers of San Diego (FHCSD), one of the 10 largest FQHCs in the United States, providing care to over 227,000 patients each year with a primary focus on uninsured, low-income and medically underserved populations. Participants included physicians, nurse practitioners, nurses, and physician assistants across the FHCSD network; clinic administrators did not participate in the study but were interviewed for their perspectives on clinic impacts.

### 2.4 Recruitment and compensation

Recruitment and compensation for clinicians to participate in the randomized control trial are fully described in a companion paper on LC&FIRP clinician outcomes (49). In brief, clinicians were recruited through email invitations to eligible FHCSD clinicians and with promotion by FHCSD medical leadership at quarterly clinician meetings. Eligible participants were FHCSD primary care physicians, physician assistants, or nurse practitioners involved in clinical care who were willing to care for patients experiencing reduced health-related quality of life and symptoms associated with Long COVID, ME/CFS, or other IACC and to participate in LC&FIRP for up to 37 months. Interested and eligible clinicians provided written informed consent and completed a baseline survey before being randomized 1:1 to the control or intervention group.

Twenty clinicians were enrolled, with participation duration varying across clinicians and a maximum individual participation of 34 months. Participation was voluntary and clinicians could withdraw at any time.

Clinicians were offered continuing medical education credit for attending each session type (upon successful completion of the respective session survey). They also received their usual hourly rate for attending weekly ECHO sessions or monthly webinars, and intervention clinicians earned their usual hourly rate to write up and present a case presentation (up to 1 h). SMEs received $400 for participation in weekly ECHO sessions only. All clinicians received their hourly rate compensation for participating in the interviews. No compensation was provided for participation in surveys.

Clinic administrators were not participants in the trial, but they were included in the initial clinician recruitment emails to inform them of the project. They were later invited to participate in qualitative interviews to provide their perspectives on clinic-wide benefits or challenges of LC&FIRP participation. Administrators were blinded to clinician trial status, however, at the time of the email invitation to participate in the qualitative interviews, the study manager informed clinic administrators which of their clinicians had participated in LC&FIRP so they could appropriately reflect on those clinicians during the interviews.

SMEs were recruited nationally across relevant clinical disciplines- including ME/CFS, pulmonology, neurology, psychiatry, cardiology, physical medicine and rehabilitation, and individuals with lived experience. Initial recruitment occurred through emails to individuals made by study-team referrals, with additional SMEs identified through professional referrals and recent scientific publications. Each weekly and monthly session included at least one specialist in physical medicine and rehabilitation, one expert with lived experience, and additional specialists selected based on the topic or case presentation. Given the breadth of topics and varying SME availability, a large SME directory was developed to ensure appropriate expertise and complete panel coverage for every weekly or monthly session.

National primary care clinicians that were invited to participate in monthly webinars and quarterly short courses were not part of the trial and are not included in this study. These broader audiences were reached nationally through dissemination by implementing partner organizations, including professional listservs, email announcements, cross-promotion through partner networks, and the LC&FIRP Project ECHO webpage. Continuing education credits were offered to encourage participation.

### 2.5 Measures

Study measures align with Klaic et al. framework assessing program implementability (34). This framework was selected as it was designed to assess the strengths and barriers of an intervention to inform future implementation, which aligned closely with the purpose of our evaluation. Authors define “implementability” of an intervention as the likelihood of adoption into routine practice across settings over time. Their measurement constructs include acceptability, feasibility, fidelity, sustainability, and scalability. We adopted these constructs, and identified specific measures appropriate to the LC&FIRP program, described below and summarized in Table 1.

**Table 1:** Constructs for assessing LC&FIRP implementation effectiveness and respective data sources.

Klaic et al. construct	Sub-topic	Unit of analysis	Description	Sources and objectivity of data
Acceptability	*Clinician engagement*	Clinicians in both arms	Number of clinicians who participated in the model	Objective: Zoom participation records
			Degree of participation by clinicians	Objective: Zoom participation records
	*Satisfaction*	Clinicians in both arms and SMEs	Self-reported intent to participate (and optimal frequency)	Subjective: Qualitative interview
			Self-reported ratings of satisfaction including: - overall satisfaction with series/ session, - willingness to participate in a future series, - if they would recommend the series to a colleague - satisfaction with engagement of lived experience experts	Subjective: Quarterly and Session surveys
	*Utility*	Clinicians in both arms	Number of clinicians who reported: - they encountered a case similar to those presented in sessions - they shared learning with colleagues to aid patient care	Subjective: Quarterly surveys
		Clinicians in both arms and SMEs	Features of the model deemed most useful	Subjective: Qualitative interviews
		Clinicians in both arms, administrators and SMEs	Perceived benefits to participants	Subjective: Qualitative interviews
Feasibility	*Clinician attrition*	Clinicians in both arms	Number of clinicians who exited or paused the study before study end	Objective: Administrative records
	*Challenges / barriers to participation*	Clinicians in both arms, administrators, and SMEs	Self-reported challenges to participation	Subjective: Qualitative interviews
Fidelity	*Adherence to study protocol*	LC&FIRP program model	Evidence of input and processes remaining consistent with what was proposed in the study protocol	Objective: Administrative records
Sustainability and Scalability	*Evidence of sustainability or scalability*	LC&FIRP program model	Plans for, or evidence of, maintenance or scaling of the LC&FIRP program after study end	Objective: Administrative records
	*Challenges to sustainability* *or scalability*	Clinicians in both arms, administrators, and SMEs	Challenges reported pertaining to sustainability and scalability of the model	Subjective: Qualitative interviews
	*Recommendations to improve sustainability or scalability*	Clinicians in both arms, administrators, and SMEs	Recommendations to improve the sustainability and scalability of the model	Subjective: Qualitative interviews

Note: The Long COVID and Fatiguing Illness Recovery Program (LC&FIRP) was a two-arm, single-blind, cluster randomized controlled trial. Intervention clinicians were invited to participate in weekly case-based interactive sessions led by national multi-disciplinary pool of subject matter experts (SMEs). Intervention and control groups were also invited to monthly webinars and quarterly online asynchronous short courses. Trial clinicians completed quarterly surveys measuring their experiences, knowledge improvement, and changes to experience as well as session-based surveys after every weekly ECHO session or monthly webinar or quarterly asynchronous online short course they attended. Qualitative interviews were conducted at study end with trial clinicians, SMEs, and clinic administrators. Zoom participation records were produced for every session.

Acceptability is explained by Klaic et al. as a measure of whether those delivering or receiving the intervention perceive it as reasonable, fair, or satisfactory. We selected three sub-constructs: clinician engagement, utility, and satisfaction. Clinician engagement was measured by the number of participants and their degree of participation. Utility of the series was measured by clinician reporting on whether they encountered a case similar to those presented in the series or shared their learning with colleagues to aid patient care, and also by soliciting participant perspectives on useful features of the series and perceived benefits of the model. Lastly, satisfaction of participants was probed directly as participant reporting of: overall satisfaction with the series or session, and satisfaction with lived experienced experts in particular, and participant willingness to attend future weekly or monthly sessions or recommend the respective series to a colleague.

Klaic et al. explains feasibility as a measure of whether the implementation is practical or viable in the relevant context, considering factors such as resources, time, and organizational support. We measured feasibility by exploring participant perceptions of challenges and barriers to accessing and participating in the educational offerings, as well as by analyzing clinician attrition.

Fidelity is described by Klaic et al. as measuring whether the model was implemented as it was planned, and/or with consistency or accuracy. For this concept we examined adherence to the study protocol by evaluating any differences in the inputs and processes planned for the LC&FIRP model in the study protocol, and how they occurred in practice.

Lastly, sustainability and scalability, as explored by Klaic et al, are constructs that help determine whether the intervention can be maintained or implemented in additional settings or populations in the future. We measured these constructs by documenting any plans for, or evidence of, sustainability and scalability and by soliciting participant perspectives on barriers to sustaining and scaling the intervention after the study end. Findings are presented across these five constructs.

### 2.6 Data collection

Session surveys were administered to trial clinicians immediately following every weekly ECHO session, monthly webinar, and quarterly short course from December 2021- October 2024, to understand participant perspectives on each session. Study staff pasted a link to the surveys in the Zoom chat at the end of every weekly ECHO session and monthly webinar, which were held on that platform. As the quarterly short courses were held asynchronously, session surveys were automatically presented to participants along with the post-session quiz. In total, 89 weekly and 34 monthly post-session surveys were administered using the REDCap and 9 quarterly short course surveys were administered via the University of Colorado web page (35).

Additionally, quarterly follow-up surveys of trial clinicians were also administered through REDCap, at baseline and at 3, 6, 9, 12, 18, 24, and 30 months post baseline, to capture clinician perspectives on the series as a whole over time. These surveys were sent to clinicians via emails from FHCSD research staff. Instruments assessed satisfaction, perceived utility, and self-reported knowledge, self-efficacy, and intention, and actual changes, to practice. We report the number of surveys completed and completion rates for weekly ECHO session and monthly webinar surveys in Supplementary Table S1. Quarterly short course survey completions are not included; too few trial clinicians completed these surveys to support analysis, so data were excluded from analysis and reporting.

Survey instruments utilized the terminology for Long COVID that was common in the scientific literature and CDC guidance at the start of data collection- “post-acute sequelae of SARS-CoV-2 infection (PASC).” (36) ME/CFS, Postural Orthostatic Tachycardia Syndrome (POTS) and other post infection fatiguing illnesses were referred to in our instruments as “other fatiguing illnesses”, a term used by CDC and others at the time (37, 38). Terminology evolved over the study period; by 2022, CDC described Long COVID as a broad term including the signs, symptoms, and conditions occurring after SARS-CoV-2 infection (39). More recently, CDC has established a broader category of IACC, which includes Long COVID, ME/CFS, and other chronic conditions associated with prior infections (40). We adopt these recent terms in our manuscript.

Semi-structured interviews were conducted with trial clinicians at study end or exit. Four clinicians declined to be contacted for interviews after trial exit, so invitations to interview were provided to 16 clinicians. SMEs were also interviewed at study end and were categorized by level of engagement: Group 1 (1 session only); Group 2 (2- 9 sessions); Group 3 (10 sessions or more). Interview guides explored satisfaction, challenges and benefits, and sustainability and scalability considerations. Clinic administrators were also interviewed at study end regarding program benefits, challenges, and sustainability and scalability. All interviews were conducted and recorded using Zoom videoconference by the primary author, and participation rates are presented in Table 2. All participating clinicians, SMEs, and clinic administrators were invited for interview via email by the primary author at study end or study exit. Participation was voluntary, and three reminders were sent to encourage participation.

**Table 2:** Summary of qualitative interviews conducted.

Interview time period	Type	Group	Invited	Interviewed	Participation rate among those invited
February 2023-October 2024	Clinicians	Control	8^a^	8	100%
		Intervention	8^a^	6	75%
		Subtotal Clinicians	16	14	88%
October -November 2024	Administrators	Control only clinicians	4	1	25%
		Intervention clinicians	4	2	50%
		Both intervention and control clinicians	3	3	100%
		Subtotal Administrators	11	6	55%
October - November 2024	SMEs	Group 3 (10 + sessions)	12	8	67%
		Group 2 (2–9 sessions)	12	5	42%
		Group 1 (1 session)	23	3	13%
		Subtotal SMEs	47	16	34%
	Total		74	36	49%

a Two control and two intervention clinicians were not invited to interview because upon exit of the trial they declined to participate in further data collection. As a result, while 88% of the clinicians who were invited to interview participated, just 14/20 (70%) of randomized trial clinicians participated in qualitative interviews.

### 2.7 Data analysis

Quantitative data analysis methods are described in the companion paper on LC&FIRP by Zuber, A. et al. (49). For this paper, the primary and secondary author quantitatively analyzed all satisfaction- and utility-related survey items for both trial groups, collapsing Likert scale item responses indicating a satisfied or favorable response (e.g., “yes” or “strongly satisfied” or “satisfied”). To test for differences between intervention arms, we followed the methods of the above-mentioned companion paper and use generalized linear models to test the effect of the intervention, while adjusting for baseline confidence in ability to treat Long COVID, the sex of the clinician, years of experience, and educational degree. We present quarterly clinician follow-up survey data in this paper and weekly ECHO session and monthly webinar survey data in Supplementary Table S2. Quarterly short course survey data is excluded from Supplementary Table S2 due to insufficient responses to support analysis.

For qualitative interviews, we produced de-identified transcriptions of the Zoom video recordings and spot-checked for accuracy, with assistance from a transcribing consultant. Transcriptions were uploaded to the NVIVO 14 software program (41), and a codebook was created using the following deductive codes: feasibility, impacts, satisfaction, barriers to sustainability and scalability, and recommendations. Qualitative analysis was conducted, following the three stage method recommended by authors Forman and Damschroeder: 1) immersion (full text review and memo-taking), 2) deduction (deductive coding, followed by secondary, inductive coding- produced through review of material coded to deductive codes and identification of new secondary sub-themes), and 3) interpretation (synthesis of findings) (42). The primary author produced all qualitative analysis and discussed results with the research team to assist in interpretation.

## 3 Results

### 3.1 Participants

Participants included 10 intervention and 10 control clinicians, and 47 SMEs. One clinician dropped out from the trial before responding to the quarterly survey, leaving 9 intervention clinicians. Six administrators at participating FHCSD clinics consented to interviews. The SME panel was not fixed across sessions. Among the 47 SMEs, 23 attended one session only (Group 1), 12 attended 2–9 sessions (Group 2), and 12 attended 10 sessions or more (Group 3), demonstrating a mix of recurring SMEs and specialists who participated more selectively according to session topic or availability.

### 3.2 Acceptability

#### 3.2.1 Clinician engagement (monthly)

Intervention and control clinician groups each had a median attendance of 13 monthly webinars (of 34 monthly webinars offered, or 38%). Attendance was stable throughout 2022 and 2023, declining in late 2023 (Figure 1). The distribution of total sessions attended by each individual was also similar between trial groups, with a few high and low attenders and a plurality of moderate attenders (Supplementary Figure S1).

**Figure 1:**
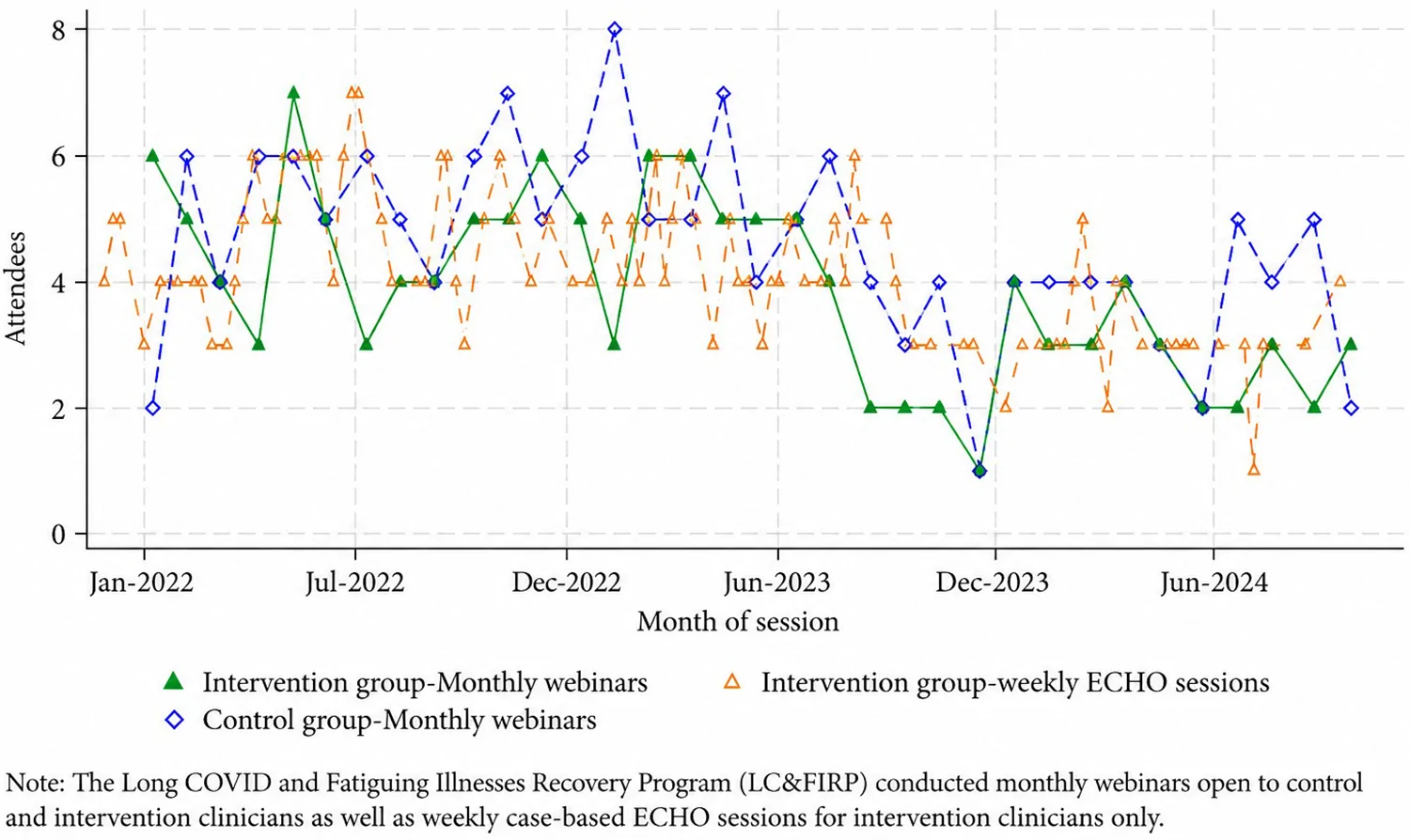
LC&FIRP monthly webinar and weekly ECHO session attendees by session date and intervention arm, Jan 2022 to Oct 2024.

#### 3.2.2 Clinician engagement (weekly)

For clinicians in the intervention group, mean and median attendance were both 37 sessions (or 42% of the 89 sessions offered), approximately 29 hours. Participation in the weekly ECHO sessions ranged from 3 sessions to 67, with 8 (of 10) intervention clinicians attending 22 sessions or more. Attendance at the weekly ECHO sessions was approximately 40% per session (4 of 10 intervention clinicians) throughout the trial (Figure 1) with no significant linear trend between week of trial and attendance.

In summary, intervention clinicians attended 50 learning sessions (webinars and ECHO sessions) on average over the course of the trial and control clinicians attended 13 sessions (webinars only) (49). Attendance by control or intervention clinician of quarterly short courses was negligible, with median attendance of zero and average attendance below one for both groups.

#### 3.2.3 Satisfaction and utility

From the quarterly surveys, satisfaction was very high for both clinician groups, as measured by their reported willingness to participate in future weekly ECHO sessions or monthly webinars, whether they recommended the weekly or monthly offerings to a colleague, and their overall satisfaction rating (97.7% for the intervention group and 91.8% for the control), averaged over the duration of the trial (Table 3). We tested treatment effects on satisfaction using our preferred model to test efficacy (50). Adjusted for baseline control variables, the intervention effect was associated with a small increase in satisfaction relative to the control group (8.1 ppt 95% CI [0.8 ppt to 15.5 ppt], t-statistic = 2.2).

**Table 3:** Percent of LC&FIRP clinicians reporting satisfaction with their respective session series in quarterly survey by intervention arm.

Item	Control group (*N* = 49 from 10 respondents)	Intervention group (*N* = 43 from 9 respondents)	Intervention effect (model-adjusted, *N* = 92)
Would you participate in a future ECHO session/ webinar/short-course like this one?	77.6%	83.7%	11.3%
Have you helped a colleague care for their patient based on information learned from these ECHO sessions/webinars/short-courses?	67.3%	69.8%	12.3%
Would you recommend this ECHO session/ webinar/short-course to a colleague?	85.7%	74.4%	−3.9%
My overall satisfaction with the ECHO session/ webinar/short-course is:	91.8%	97.7%	8.1%^a^
Have you encountered a case similar to one presented during a previous ECHO session/ webinar/short-course?	79.6%	97.7%	13.4%^a^

The Long COVID and Fatiguing Illness Recovery Program supported the participation of 20 clinicians to monthly webinars comprised of didactic education and peer exchange (as well as quarterly asynchronous online short courses), and invited intervention clinicians to additionally attend weekly, case-based ECHO learning sessions.

a The indicates that the intervention group differed significantly from the control group at alpha = 0.05. Items were coded to indicate the proportion satisfied. Future participation was coded as positive if definitely yes, probably yes. Satisfaction was coded as positive if response was strongly satisfied or satisfied. Helping a colleague and encountering a similar case were both yes/no items. Recommendation was considered positive if definitely yes or probably yes. Adjusted model uses population averaged panel model with exchangeable in-group correlation structure, adjusted for baseline confidence, sex of clinician, experience, and degree of clinician.

Utility of each series was also high for both groups, as measured by whether they encountered a case similar to those presented in the weekly ECHO session or monthly webinar and they shared learning from the modality with colleagues to aid patient care (Table 3). To test whether these high rates of satisfaction and utility were increasing or decreasing over the course of the trial, we ran our preferred model but replaced month binary indicators with a single linear variable capturing the month of the trial. This variable did not reach statistical significance in any of the five outcomes. Overall satisfaction and encountering similar cases were both significantly higher for the intervention group. Session-based surveys showed the same trend of more favorable ratings by intervention clinicians, even when the session being evaluated was the same between clinician groups (Supplementary Table S2). Satisfaction and utility pertaining to quarterly short courses could not be assessed given the negligible participation of all trial clinicians.

#### 3.2.4 Qualitative perspectives

##### 3.2.4.1 High satisfaction with the series and key features

In interviews, both clinician groups and SMEs expressed high satisfaction with the sessions they participated in and also did not differ in their willingness to participate in the respective weekly or monthly sessions in the future. Clinicians across both groups reported they liked the concise format of the sessions, and the opportunity to learn from peers, and they viewed the practical approaches to patient care as highly applicable to their work.

“The format is nice …because I have a short attention span, one hour is perfect for me as opposed to sitting through three days of conference where you are brain dead after the first day” (intervention clinician)

In contrast, three clinicians (two control, one intervention) reported they would not participate in the future due to changed job positions, competing priorities, or the lack of meaningful treatment updates warranting attendance.

SMEs further valued the structure of both types of sessions, the professionalism of the organizing team and moderators, and productive use of the chat function during the sessions.

“The structure itself…was very beneficial… you knew in advance what the topic would be…and the moderation was designed in a way where it was very productive” (SME).

All clinicians reported a high degree of satisfaction with the participation of lived experience experts in both session types, who helped them to enhance empathy, patient listening, and patient communication. The clinicians gained “more of an appreciation for the huge breadth of (symptom) severity”, “more empathy and understanding”, and more flexibility in how they prescribed interventions to patients based on the suggestions of these experts.

“They were very good about knowing the intricacies of the system, knowing (about) resources and online support groups” (intervention clinician).

##### 3.2.4.2 Benefits to confidence, perceived quality of care, and professional networks

Clinicians reported the most important benefits to participation in either session type were improvements to their confidence and perceived quality of care for patients with Long COVID, ME/CFS and/or IACC, as well as the opportunity to learn and share with other clinicians. Many clinicians from both groups reported feeling “lost” or “anxious” about treating these patients before the LC&FIRP series and reported the series gave them a greater awareness of diagnostic tools and new therapeutics and ideas for what to try with their patients, based on the approaches of other clinicians and lived experience experts. This improved their confidence managing patients in the absence of clinical guidelines or evidence-based approaches.

“(Before the series) when I saw (a patient with suspected Long COVID) I thought “I don't know how to handle this, I don't know how to help you. I'm going to try and get you to a specialist and not [to] come back to primary care…because I feel so overwhelmed by it….(LC&FIRP) helped me break down the steps, try interventions, bring them back more often, feel more confident in just working through it with them” (intervention clinician).

Clinicians in both groups also reported improved acceptance and compassion for their patients, as well as enhanced patient listening and communication skills.

“I was better informed, I could answer questions better for my patient, I could honestly answer questions. I didn't feel like I had to be evasive or give the patient false hopes, false information” (control clinician).

SMEs reported acquiring new knowledge and skills from the series, mostly from other SMEs in other disciplines. The WhatsApp chat itself was a popular resource; SMEs reported it reduced their sense of isolation working in Long COVID, enabled discussion on advancements, other podcasts and scientific resources, and helped them to locate specialists for their own patient referrals. The SMEs reported these relationships led to “spin-offs” of academic or clinical collaborations between SMEs. An unexpected benefit of the series among SMEs was the improvement in their own confidence and perceived quality of care of patients with Long COVID, ME/CFS and other IACC, which was not an explicit objective of the trial.

Administrators of clinics with participating clinicians from both trial conditions demonstrated a superficial awareness of the trial. However, they observed improvements to clinician confidence and to the perceived quality and efficiency of patient care in their settings. Administrators also observed their participating clinicians sharing lessons learned from the trial with non-trial clinicians, such as in “curbside patient discussions” or routine meetings. Administrators overseeing clinicians in both trial conditions reported no differences in perceived benefits to the clinic between the intervention and control conditions.

Satisfaction and perceived utility, including any benefits to participation, could not be assessed among trial clinicians or quarterly short courses, due to their negligible participation in that modality.

### 3.3 Feasibility

#### 3.3.1 Clinician attrition

Fifty percent of the 20 clinicians recruited remained in the study for the full intended duration. Six clinicians dropped out of the trial, one at enrollment and one at the 5-month time period, of which five were intervention clinicians. Four clinicians paused participation (i.e., no new referrals or participation in study activities with an intent to reengage), of which three were control clinicians. The rationale for exit was primarily a departure from FHCSD (due to new employment or retirement) and time commitment, while the rationale for pausing was due to transfer to other departments within FHSCD, limited availability and heavy caseload.

#### 3.3.2 Qualitative perspectives

##### 3.3.2.1 No hurdles in physical access to synchronous sessions; video recordings difficult to find

Clinicians and SMEs reported experiencing no hurdles to physically accessing or participating in the weekly ECHO monthly or monthly webinars. However, many SMEs found the video recordings and materials difficult to locate, as they could not be searched by topic; however, they were easy to use once located.

##### 3.3.2.2 Time commitment is a major barrier

All clinicians and SMEs cited time commitment as the major challenge to participate regularly in weekly ECHO sessions and monthly webinars. Intervention clinicians found the weekly cadence too onerous over the 30-month duration of the trial, while all clinicians and SMEs cited challenges attending all types of sessions due to conflicts with other meetings, the demands of patient care, administrative duties, or mealtimes. While most clinicians appreciated the sessions offered at lunchtime (versus during patient care slots), they still struggled with transitioning between patient care windows, participating while eating, and losing time that would otherwise be spent on important administrative tasks.

“I have a lot of other hats in this organization, and I've got all these fires that I'm putting out and having that expectation to be there weekly was simply a bridge too far” (intervention clinician).

“I really struggled to finish clinic and log in on time. I would race from seeing a patient, go to the bathroom, struggle to get logged on, then rush on the other side to get to my patient visit” (intervention clinician).

##### 3.3.2.3 Case preparation workload is onerous

Intervention clinicians were most concerned about the time required to prepare case presentations for weekly ECHO sessions, a task that was not required of control clinicians. This workload increased as clinicians exited the trial, as sessions had to be covered by the remaining clinicians.

“There were a very small number of us actually on this interventional side…by the end it was maybe four or five…. there was an expectation that we would be presenting a case every week… it wasn't realistic. It was just too much” (intervention clinician).

##### 3.3.2.4 Administrative approval limits SME participation

One challenge experienced primarily by SMEs was the lack of administrative approval to participate in the live, synchronous sessions (i.e., weekly ECHO sessions or monthly webinars). The reasons provided were that the sessions interfered with their patient slots or that administrators were skeptical of Long COVID as a condition.

##### 3.3.2.5 Patient population has unique needs

Multiple control clinicians attending monthly webinars noted that caring for patients with Long COVID, ME/CFS, and other IACC requires more time per visit, posing a challenge to trial participation.

“They tend to be very long appointments and we only get 20 min, so I often was spending 40 and then just scrambling to make up the rest of the day” (control clinician).

Feasibility considerations for quarterly short courses could not be assessed given the negligible participation by trial clinicians in those course offerings.

### 3.4 Fidelity

Table 4 compares planned and implemented program inputs and processes for weekly ECHO session, monthly webinars and quarterly short courses. Fidelity was maintained in funding, partners, clinician recruitment, and the monthly webinars and quarterly short courses provided to both trial groups. However, fewer weekly ECHO sessions and case presentations were conducted than were planned in the study protocol. FHCSD staff reported that some weekly ECHO sessions were cancelled due to scheduling issues related to a shortage of facilitators, last-minute cancellation of didactic speakers, and holidays. The target of having a case presentation for each session was not met because clinicians lacked time to complete the form, pull patient data, prepare questions, and prepare their presentations.

**Table 4:** Summary of fidelity of LC&FIRP implementation.

Category	Planned in study protocol	Achieved in implementation	Percent achieved
Inputs			
Funding	$3 million	$3 million	100%
Partnerships	FHCSD, University of Colorado, University of Washington, Ata Health	FHCSD, University of Colorado, University of Washington, Ata Health	100%
Patients recruited	856	590	69%
Clinicians recruited	20	20	100%
Processes			
Number of weekly ECHO sessions (intervention condition only)	136	89	65%
Number of monthly webinars	34	34	100%
Number of quarterly short courses	10	9	90%
Number of case presentations (intervention condition only)	136	52	38%
Quarterly FCHSD intervention clinician check-in meetings	0	8	N/A

The Long COVID and Fatiguing Illness Recovery Program (LC&FIRP) was a two-arm, single-blind, cluster randomized controlled trial. Intervention clinicians were invited to participate in weekly case-based ECHO learning sessions led by national multi-disciplinary pool of subject matter experts (SMEs). In weekly ECHO sessions, intervention clinicians presented cases of patients for input by a panel of multidisciplinary specialists. Intervention and control groups were also invited to monthly webinars and quarterly asynchronous online short courses.

Another departure from planned intervention processes was the introduction in October 2022 of quarterly check-in meetings with intervention clinicians, hosted by the FHCSD study team. The quarterly check-ins were requested by participating clinicians to discuss how to manage and care for patients in the program, including how to utilize feedback from SMEs, access patient resources, and use the referral network. Eight check-ins were held, starting October 27, 2022 and ending on June 20, 2024. They were led by a FHCSD facilitator who was also a clinician.

### 3.5 Sustainability and scalability

#### 3.5.1 Evidence of maintenance or scaling at study end

Weekly ECHO sessions and monthly webinars were both sustained through new funding by the U.S. CDC and the Schmidt Initiative for Long COVID (SILC). After the formal study period, the weekly ECHO sessions were opened to primary care clinicians nationwide and globally treating patients with Long COVID, ME/CFS and other IACC, and divided into two cohorts, each meeting monthly, rather than weekly. The monthly webinars maintained the original format. The quarterly short courses were not continued.

#### 3.5.2 Qualitative perspectives

##### 3.5.2.1 Time constraint for participation and unique patient population care

Multiple clinicians from both trial conditions and SMEs cited the time commitment to participate as the chief barrier to the sustainability and scalability of weekly ECHO sessions and monthly webinars, with intervention clinicians emphasizing the extra workload related to case preparation. Participants also referenced the additional time needed for this population of patients.

“The time commitment …is a challenge that's twofold because it's a time commitment to go to the meetings and to learn and to write the case studies, but it's also a time commitment to commit to taking on those challenging patients”. (SME)

##### 3.5.2.2 Clinician fatigue and reduced demand for COVID-19

Several SMEs emphasized concern that sustaining and scaling weekly ECHO sessions and monthly webinars will become more difficult in the future due to clinician “fatigue” with COVID-19-related learning*.*

“Fatigue plays a role. I've been involved in a lot of different Long COVID things and they tend to just taper off and trickle out over time…maybe it loses some of its novelty.” (control clinician)

Control clinicians also speculated that patient demand for Long COVID care may decrease due to a perception of lowered risk, which may reduce clinician interest in continued COVID-19 learning.

##### 3.5.2.3 Funding and administrative support

SMEs further identified funding for program delivery and participant incentives as another barrier to sustainability and scalability of the weekly and monthly sessions. As one noted: *“Without the (study) funding, I don't think clinicians would join every week”* (SME). Another stated:

“The only limitation I see for this program is resources. The demand is absolutely there” (SME).

Clinicians emphasized the need for protected time slots that do not conflict with patient care. Clinic administrators raised concerns that administrators in new settings may not provide enough leadership buy-in or support for their SMEs or clinicians to participate.

##### 3.5.2.4 Recommendations for improved sustainability and scalability

To improve the sustainability and scalability of the weekly and monthly sessions, clinicians and SMEs made key recommendations that can be summarized in the following list (not in ranked order): (1) rename the series of weekly ECHO sessions and monthly webinars to focus on “post-infection illnesses” (i.e., IACC) more broadly to generate more interest and be inclusive of emerging conditions; (2) reduce weekly ECHO sessions to biweekly or monthly intervals; (3) adjust compensation practices be to better reflect the workload (i.e., diminish honoraria for panelists and increase it for case presenters); (4) shorten session duration to 45–50 min, to enable clinician transitions; (5) place greater emphasis on interactive, case-based discussion in monthly webinars; (6) reduce focus on academic content without clear clinical application; (7) maintain lived experience expert participation; (8) provide regular updates to clinic administrators on program progress, costs, and patient care benefits.

## 4 Discussion

Our study assessing the implementation effectiveness of a multi-modal program of ECHO case-based learning coupled with broader educational modalities for the management of Long COVID, ME/CFS and other IACC demonstrates high acceptability among participating clinicians and among SMEs and administrators we interviewed for weekly ECHO sessions and monthly webinars. However, sustaining full engagement among clinicians in all offerings over the 30-month trial was a challenge, due to feasibility constraints, and implementation fidelity was also mixed. Participation in quarterly short courses by all trial clinicians was negligible and therefore neither quantitative nor qualitative data could be analyzed. Early sustainability and scalability of the weekly ECHO sessions and webinars were observed, but barriers to future success in this area were identified.

The acceptability of the ECHO weekly sessions– when measured by clinician reported satisfaction, willingness to participate in the future, and likelihood to recommend sessions to other colleagues–was consistently high throughout the trial, consistent with the ECHO literature (7, 43). The acceptability of the monthly webinars was consistent in the same measures. Importantly, clinicians from both groups reported substantial professional and clinical benefits from participation (with no observable differences between groups), including perceived improvements to patient care. These findings were corroborated by SMEs and clinic administrators and are supported by quantitative results reported in a companion paper by Zuber et al. 2026 (unpublished), which demonstrated improvements in knowledge, confidence, and practice among participating clinicians in both trial groups.

Participants most valued weekly ECHO sessions or monthly webinars that promoted peer and SME interaction– including lived experience experts- and practical diagnostic and treatment guidance. These features reduced clinician uncertainty and supported care decision-making in the absence of established clinical guidelines. Participants unfavorably viewed sessions that were academically oriented or presented emerging research without practical applications. This finding reinforces the model's suitability for providing education in conditions that lack evidence-based guidelines, consistent with prior ECHO studies during COVID-19 (7, 8), and points to the importance of focusing future sessions on practical knowledge and skills (over academic areas of interest).

In terms of participation or engagement, however, the acceptability is lower. Clinicians attended a meaningful number of sessions, but considerably less than offered (no *a priori* target per individual was established). Intervention clinicians attended 42% of the weekly ECHO sessions offered, on average, while intervention and control clinicians each attended 38% of the monthly webinars. They also exited the trial altogether at a higher rate than control clinicians (5 interventions clinicians versus 1 control clinician). Low trial clinician engagement with quarterly short courses—which led to it being dropped as a unit of analysis from this study due to insufficient data- suggests this modality offered the least value to clinicians already attending weekly and monthly offerings. It is also not a common feature of ECHO learning models (20, 44, 45). However, the quarterly courses reached a broad national audience (927 registrants and 516 completers), which make them a robust dissemination platform to clinicians and other professionals not attending the more routine offerings.

Our feasibility findings help explain the more limited participation in the weekly ECHO sessions and monthly webinars. All trial clinicians described competing clinical and administrative duties, scheduling conflicts, limited time, and difficulty transitioning between sessions and clinical duties as barriers to participation in either modality. Intervention clinicians additionally described the weekly cadence over 30 months as burdensome and identified the time required to prepare patient cases for weekly ECHO sessions as a challenge. These findings are consistent with participation challenges cited in a qualitative review of five case-based ECHO virtual communities of practice by Agley, J. et al. (45).

Notably, our unpublished companion study by Zuber et al., 2026 found that the estimated clinician exposure to weekly ECHO sessions associated with maximal self-reported intervention effects was 74 sessions for confidence and changes to practice and 65 sessions for knowledge; this is less than the 132 total sessions offered by LC&FIRP. Also considering the gradual decrease in participation of clinicians towards the trial end, we recommend that future implementation comprise of a shorter intervention period that provides sufficient exposure to achieve benefit while reducing the participation burden. Feasibility can be further improved by reducing session length to 45–50 min and using alternative models of case preparation.

Several inputs and processes for both trial conditions were implemented with fidelity to the study plan, including funding, partners, clinician recruitment, monthly webinars, and quarterly short courses. However, weekly ECHO sessions and case presentations—a central feature of the intervention condition- were lower than planned. This was partially explained by intervention clinician feedback that case presentations were more difficult to produce by clinicians as the series progressed, as increasing clinician attrition from the trial increased the case presentation workload for remaining clinicians. These findings suggest that future implementation should establish a case-presentation cadence that can be sustained within expected levels of clinician participation and potentially increase the compensation or other incentives provided for this task.

While the ECHO model is thought to be sustainable in many settings due to its lower costs and improved accessibility relative to in-person educational approaches (8, 21), the LC&FIRP model may struggle to recruit and retain clinicians in the future, in light of increasing clinician fatigue with COVID-19, perceptions of decreased patient demand for post-COVID services, and increased resources required for the complementary educational offerings. The clinician recommendation to rename the series to IACC could broaden interest and participation. The incentive payments to clinicians and SMEs also increases the cost to sustain the model as compared to other ECHO models; aligning compensation more closely to time burden and considering alternate forms of incentives is recommended.

Scalability is infrequently examined in ECHO evaluations, and thus our study offers initial insights that may inform future evaluations (20, 26, 45). A potential scaling strategy is to maintain the three components as a combined program model, whereby more frequent case-based ECHO sessions among a small group of clinicians generate practical knowledge that can be that disseminated through less frequent webinars and asynchronous online short courses for broader audiences. The conversion of the LC&FIRP weekly ECHO sessions to a monthly cadence after the trial ended (through new funding) provides some evidence that monthly ECHO sessions may be more feasible for sustained implementation. Under such a model, webinars and asynchronous online short courses could be offered less frequently to extend the reach of knowledge generated through the ECHO sessions to broader audiences.

At the same time, although this approach may be cost efficient, findings from the companion paper in this journal by Zuber, A. et al, 2026 (unpublished) suggest a trade-off. Clinicians participating in the webinars and asynchronous online short courses may experience lower knowledge improvements and confidence gains than participants in the ECHO sessions. Given the low participation of trial clinicians in the asynchronous quarterly short courses, this component is best positioned as optional or fee-based continuing medical education targeted to clinicians who are not otherwise participating in ECHO sessions or webinars (45).

### 4.1 Limitations

Our study faced some limitations. First, we conducted our study within a single FQHC network in the San Diego area, reducing generalizability to other clinician populations. Second, voluntary participation in interviews and surveys is subject to selection bias (i.e., may overrepresent the participants with higher satisfaction). We did achieve a high participation rate (88%) in qualitative interviews among clinicians invited to interview, and they represent the most important source of information for the study. At the same time, four clinicians declined further data collection after trial exit and were therefore not invited to interview. As a result, clinicians that participated in the interviews represented 70% (14/20) of all randomized clinicians. These clinicians who remained engaged in the study may have had different experiences with LC&FIRP than those who withdrew or did not participate in interviews, potentially biasing qualitative findings toward the experiences of more engaged clinicians. We also acknowledge the interview participation rate was particularly low among SMEs attending one session only, likely resulting in some bias towards SMEs that were more substantially engaged in the sessions. To help counteract selection bias, we report not just majority but also minority perspectives (i.e., “negative case reporting”) (46). Lastly, we did not set *a priori* thresholds for feasibility and fidelity measures, which is an emerging practice in implementation science that can establish a more objective and consensus-driven approach to measuring implementation effectiveness (47).

## 5 Conclusion

LC&FIRP, which couples weekly case-based ECHO virtual community of practice sessions with monthly webinars and quarterly asynchronous online short courses for primary care clinicians of patients with Long COVID, ME/CFS, and other IACC has demonstrated evidence of satisfaction, utility, and self-reported benefits among participating clinicians, SMEs, and clinic administrators. The time commitment and case preparation workload for participants are factors that jeopardize the program's ongoing feasibility. Continuation of the ECHO sessions and webinars after the trial marks early success in sustainability and scalability; however, reduced clinician interest and growing fatigue with COVID-19 learning and ongoing funding requirements pose constraints. We offer recommendations for adaptation to meet these challenges and continue this bundle of implementation approaches to clinician learning, with potential expansion to other complex chronic conditions for which clinical guidelines and evidence-based practice are emerging.

## Data Availability

The raw data supporting the conclusions of this article will be made available by the authors, without undue reservation.
