# Supplementary material for: Implementation effectiveness of the Long COVID and Fatiguing Illnesses Recovery Program (LC&FIRP): a multimodal Extension for Community Healthcare Outcomes (ECHO) model for clinician learning
Source: Front Health Serv. 2026 Oct 23;6:1912278. doi: 10.3389/frhs.2026.1912278 (PMC13739702; doi:10.3389/frhs.2026.1912278)
Supplement: Supplementary file 1 [file Datasheet1.docx]

# Supplementary Materials

**Researcher Characteristics and Reflexivity:**

The primary investigator is a female public health professional with a doctoral degree in Public Health Leadership, based in the United States. Her prior experience in qualitative research, workforce development, and in evaluating ECHO models for public health, informed selection of study methods and provided experience to properly execute the methods. The P.I. is not a clinician and therefore made sure that all study instruments were carefully reviewed by clinician members of the research team for accuracy. The P.I. further acknowledged her position as an outsider to clinical practice in her interviews with clinicians and administrators, to assist in the interpretation of comments and feedback that assume a clinical background or context. The P.I. further shared study findings in an interactive fashion with clinician members of the team for improved interpretation and validation.

**Supplementary Table 1. Number of surveys, responses, and survey participation rates by type of survey**

|  | **Quarterly**  **feedback survey** | **Monthly**  **post-session survey** | **Weekly**  **post-session survey** |
| --- | --- | --- | --- |
| Trial condition | Intervention and control | Intervention and control | Intervention only |
| Number of distinct surveys administered | 8 | 34 | 89 |
| Number of surveys administered to individuals | 152 | 289 | 374 |
| Survey completions | 92 | 96 | 145 |
| Survey completion rate | 61% | 33% | 39% |

Note: Survey completion was defined as a response to at least one survey item. Quarterly feedback survey results are reported in Table 3; monthly and weekly post-session survey results are reported in Supplementary Table 2. Quarterly asynchronous short course survey completions and completion rates are not presented in this table, due to the fact responses to those surveys by trial clinicians were too few to support analysis and were therefore excluded from this evaluation.

**Supplementary Table 2. Summary data from post-session evaluations of weekly ECHO sessions and monthly webinars**

|  | **Weekly Survey (N=145)** | **Monthly Survey**  **(N=96)** | | |
| --- | --- | --- | --- | --- |
| **Item** | **Intervention**  **Clinicians** | **Both Clinician Groups** | **Control**  **Clinicians** | **Intervention**  **Clinicians** |
| Knowledge about subject before session (% Extremely or very knowledgeable)* | 10% | 11% | 3% | 24% |
| Knowledge about subject after session (% Extremely or very knowledgeable) | 34% | 30% | 25% | 39% |
| How relevant was the session (% excellent or very good) | 73% | 78% | 83% | 70% |
| How well did the session deliver (% excellent or very good)* | 82% | 77% | 69% | 91% |
| How well were the stated objected met (% excellent or very good)* | 80% | 77% | 68% | 94% |
| Had opportunities to ask questions (% excellent or very good) | 83% | 76% | 73% | 82% |
| The organization of the presentation (% excellent or very good)* | 83% | 80% | 73% | 94% |
| The pace of the presentation (% excellent or very good)* | 83% | 78% | 69% | 94% |
| The presenter's clarity (% excellent or very good) | 83% | 80% | 76% | 88% |
| Was there commercial bias in the presentation (% no) | 99% | 100% | 100% | 100% |
| Was the source of evidence presented (% yes) | 99% | 98% | 98% | 97% |
| Will you use what you learned (% yes, probably yes, or definitely yes) | 98% | 97% | 97% | 97% |
| Would you recommend the session (% yes, probably yes, or definitely yes) | 99% | 100% | 100% | 100% |
| I intend to apply the knowledge and/or skills I have acquired (% yes, probably yes, or definitely yes) | N/A | 94% | 90% | 100% |
| I am better able to communicate with other members of a multidisciplinary team (% agree/strongly agree)* | N/A | 80% | 71% | 94% |
| I am better able to discuss how teamwork can contribute (% agree/strongly agree)* | N/A | 82% | 75% | 94% |
| Note: The percentage with a favorable response is shown for each item. *Indicates that rejection of equality between control and intervention group at 95% CI. Only the intervention group was given the weekly-session surveys. The Long COVID and Fatiguing Illness Recovery Program was a two-arm, single-blind, cluster randomized controlled trial. Intervention clinicians were invited to participate in weekly case-based interactive sessions led by national multi-disciplinary pool of subject matter experts (SMEs). Intervention and control groups were also invited to monthly webinars and quarterly online asynchronous short courses. Trial clinicians completed quarterly surveys measuring their experiences, knowledge improvement, and changes to experience as well as session-based surveys after every weekly, monthly and quarterly asynchronous short course they attended. | | | | |

**Supplementary Figure 1. Cumulative attendance at monthly webinars from Jan 2022-Oct 2024, for all trial clinicians**

Note: The Long COVID and Fatiguing Illness Recovery Program was a two-arm, single-blind, cluster randomized controlled trial. Intervention clinicians were invited to participate in weekly case-based ECHO learning sessions led by national multi-disciplinary pool of subject matter experts (SMEs). Intervention and control groups were also invited to monthly webinars and quarterly asynchronous short courses.

**Clinician Baseline Survey**

Please complete the following baseline survey.

(Publication Note: *This was administered to all trial clinicians. PASC was used to refer to what we later called Long COVID*).

**Baseline**

| **First Name*** | ________________________________ |
| --- | --- |
| **Last Name:*** | ________________________________ |

**Demographics**

| **What is your ethnicity?*** | ☐ Hispanic ☐ Non-Hispanic ☐ Prefer not to answer |
| --- | --- |
| **Which race(s) best describes you? Please select all that apply.*** | ☐ Alaksa Native ☐ American Indian ☐ Asian ☐ Black ☐ Hawaiian ☐ Middle Easter/ Arab ☐ Multi-racial ☐ Pacific Islander ☐ White ☐ Other ☐ Prefer not to answer |
| **What is your date of birth?*** | ________________________________ |
| **What is your current gender identity?*** | ☐ Man ☐ Woman ☐ Transgender female or trans woman ☐ Transgender male or trans man ☐ Nonbinary genderqueer or genderfluid ☐ A gender identity not listed here ☐ Prefer not to answer |
| **What is your current relationship status?*** | ☐ Single or casually dating ☐ In a committed relationship or engaged ☐ Living a marriage like relationship ☐ Married ☐ Separated ☐ Divorced ☐ Widowed |
| **Including yourself, how many people- adults and children- currently live or stay in your household?*** | ________________________________ |
| **How many people under 18 years old currently live or stay in your household?*** | ________________________________ |

**Clinical Role and Confidence**

| **What is your title or clinical role?*** | ________________________________ |
| --- | --- |
| **What credentials do you hold?*** | ________________________________ |
| **How many years have you been working as a clinician?*** | ☐ 1-5 years ☐ 6-10 years ☐ 10-15 years ☐ 15+ years |
| **I am confident in my ability to identify tools and methods for PASC care.*** | ☐ Strongly Disagree ☐ Disagree ☐ Neutral ☐ Agree ☐ Strongly Agree |
| **I am confident in my ability to diagnose patients with PASC.*** | ☐ Strongly Disagree ☐ Disagree ☐ Neutral ☐ Agree ☐ Strongly Agree |
| **I am confident in my ability to screen for co-morbidities of PASC.*** | ☐ Strongly Disagree ☐ Disagree ☐ Neutral ☐ Agree ☐ Strongly Agree |
| **I am confident in my ability to generate a patient-specific treatment plan for patients with PASC.*** | ☐ Strongly Disagree ☐ Disagree ☐ Neutral ☐ Agree ☐ Strongly Agree |
| **I am confident in my ability to implement a patient-specific treatment plan for patients with PASC.*** | ☐ Strongly Disagree ☐ Disagree ☐ Neutral ☐ Agree ☐ Strongly Agree |
| **I am confident in my ability to select appropriate special or diagnostic tests for PASC patients.*** | ☐ Strongly Disagree ☐ Disagree ☐ Neutral ☐ Agree ☐ Strongly Agree |
| **I am confident in my ability to interpret special or diagnostic test results for my PASC patients.*** | ☐ Strongly Disagree ☐ Disagree ☐ Neutral ☐ Agree ☐ Strongly Agree |
| **I am confident in my ability to provide appropriate patient education about their PASC condition.*** | ☐ Strongly Disagree ☐ Disagree ☐ Neutral ☐ Agree ☐ Strongly Agree |
| **I am confident in my ability to educate clinic staff about PASC care.*** | ☐ Strongly Disagree ☐ Disagree ☐ Neutral ☐ Agree ☐ Strongly Agree |
| **I am confident in my ability to address PASC patients using a multidisciplinary approach.*** | ☐ Strongly Disagree ☐ Disagree ☐ Neutral ☐ Agree ☐ Strongly Agree |
| **I am confident in my ability to assess and manage concerns that PASC patients face.*** | ☐ Strongly Disagree ☐ Disagree ☐ Neutral ☐ Agree ☐ Strongly Agree |
| **I am confident in my ability to assess severity of disease in patients with PASC.*** | ☐ Strongly Disagree ☐ Disagree ☐ Neutral ☐ Agree ☐ Strongly Agree |
| **I am confident in my ability to deal with complex cases of PASC.*** | ☐ Strongly Disagree ☐ Disagree ☐ Neutral ☐ Agree ☐ Strongly Agree |

**Clinician Quarterly Follow-up Survey	(Control clinicians)**

Please complete this 3 month follow-up survey.

(*Publication note:* *this* *survey was administered to control clinicians. PASC was used to refer to what we later called Long COVID).*

**Clinician 3M**

| **1. First Name*** | ______________________________ |
| --- | --- |
| **2. Last Name*** | ______________________________ |

**Clinical Knowledge**

| **3. My knowledge of appropriate PASC diagnosis has improved as a result of participation in the session. *** | ☐ Strongly Disagree ☐ Disagree ☐ Neutral ☐ Agree ☐ Strongly Agree |
| --- | --- |
| **4. My knowledge of effective PASC care and treatment has improved as a result of participation in the session.*** | ☐ Strongly Disagree ☐ Disagree ☐ Neutral ☐ Agree ☐ Strongly Agree |
| **5. My knowledge of approaches for complex cases of PASC has improved as a result of participation in these sessions.*** | ☐ Strongly Disagree ☐ Disagree ☐ Neutral ☐ Agree ☐ Strongly Agree |

**Clinical Confidence**

| **6. I am confident in my ability to identify tools and methods for PASC care.*** | ☐ Strongly Disagree ☐ Disagree ☐ Neutral ☐ Agree ☐ Strongly Agree |
| --- | --- |
| **7. I am confident in my ability to diagnose patients with PASC.*** | ☐ Strongly Disagree ☐ Disagree ☐ Neutral ☐ Agree ☐ Strongly Agree |
| **8. I am confident in my ability to screen for co-morbidities of PASC.*** | ☐ Strongly Disagree ☐ Disagree ☐ Neutral ☐ Agree ☐ Strongly Agree |
| **9. I am confident in my ability to generate a patient-specific treatment plan for patients with PASC.*** | ☐ Strongly Disagree ☐ Disagree ☐ Neutral ☐ Agree ☐ Strongly Agree |
| **10. I am confident in my ability to implement a patient-specific treatment plan for patients with PASC.*** | ☐ Strongly Disagree ☐ Disagree ☐ Neutral ☐ Agree ☐ Strongly Agree |
| **11. I am confident in my ability to select appropriate special or diagnostic tests for PASC patients.*** | ☐ Strongly Disagree ☐ Disagree ☐ Neutral ☐ Agree ☐ Strongly Agree |
| **12. I am confident in my ability to interpret special or diagnostic test results for my PASC patients. *** | ☐ Strongly Disagree ☐ Disagree ☐ Neutral ☐ Agree ☐ Strongly Agree |
| **13. I am confident in my ability to provide appropriate patient education about their PASC condition. *** | ☐ Strongly Disagree ☐ Disagree ☐ Neutral ☐ Agree ☐ Strongly Agree |
| **14. I am confident in my ability to educate clinic staff about PASC care.*** | ☐ Strongly Disagree ☐ Disagree ☐ Neutral ☐ Agree ☐ Strongly Agree |
| **15. I am confident in my ability to address PASC patients using a multidisciplinary approach. *** | ☐ Strongly Disagree ☐ Disagree ☐ Neutral ☐ Agree ☐ Strongly Agree |
| **16. I am confident in my ability to assess and manage concerns that PASC patients face.*** | ☐ Strongly Disagree ☐ Disagree ☐ Neutral ☐ Agree ☐ Strongly Agree |
| **17. I am confident in my ability to assess severity of disease in patients with PASC. *** | ☐ Strongly Disagree ☐ Disagree ☐ Neutral ☐ Agree ☐ Strongly Agree |
| **18. I am confident in my ability to deal with complex cases of PASC.*** | ☐ Strongly Disagree ☐ Disagree ☐ Neutral ☐ Agree ☐ Strongly Agree |

**Satisfaction**

| **19. Have you encountered a case similar to one presented during a previous webinar? *** | ☐ Yes ☐ No |
| --- | --- |
| **20. My overall satisfaction with the webinar is: *** | ☐ Strongly Satisfied ☐ Satisfied ☐ Neutral ☐ Dissatisfied ☐ Strongly Dissatisfied |
| **21. Would you recommend this webinar to a colleague?*** | ☐ Definitely Not ☐ Probably Not ☐ Possibly ☐ Probably Yes ☐ Definitely Yes |
| **22. Would you participate in a future webinar like this one?*** | ☐ Definitely Not ☐ Probably Not ☐ Possibly ☐ Probably Yes ☐ Definitely Yes |

**Changes to Practice**

| **23. Have you helped a colleague care for their patient based on information learned from these webinars?*** | ☐ Yes ☐ No |
| --- | --- |
| **24. As a result of participating in the monthly webinar: I have a better understanding of further monitoring and follow-up plan.*** | ☐ Strongly Disagree ☐ Disagree ☐ Neutral ☐ Agree ☐ Strongly Agree |
| **25. As a result of participating in the monthly webinar: I developed more appropriate treatment plans. *** | ☐ Strongly Disagree ☐ Disagree ☐ Neutral ☐ Agree ☐ Strongly Agree |
| **26. As a result of participating in the monthly webinar: I have a better understanding of underlying disease process. *** | ☐ Strongly Disagree ☐ Disagree ☐ Neutral ☐ Agree ☐ Strongly Agree |
| **27. As a result of participating in the monthly webinar: I have a better understanding of the role of specialist referral. *** | ☐ Strongly Disagree ☐ Disagree ☐ Neutral ☐ Agree ☐ Strongly Agree |
| **28. As a result of participating in the monthly webinar: I was able to more independently manage my PASC patients without referral to a specialist. *** | ☐ Strongly Disagree ☐ Disagree ☐ Neutral ☐ Agree ☐ Strongly Agree |
| **29. As a result of participating in the monthly webinar: I was not able to apply what I learned. *** | ☐ Strongly Disagree ☐ Disagree ☐ Neutral ☐ Agree ☐ Strongly Agree |
| **30. As a result of participating in the monthly webinar: I was able to apply specialist recommendations to patient care that I learned in this series. *** | ☐ Strongly Disagree ☐ Disagree ☐ Neutral ☐ Agree ☐ Strongly Agree |
| **31. As a result of participating in the monthly webinar: I made other changes to my practice.*** | ___________________________________ ___________________________________ |

**Clinician Quarterly Follow-up Survey**

Please complete this 3 month follow-up survey.

(*Publication note: This* *survey was administered to intervention clinicians. “Tele-ECHO” was used to describe the weekly sessions at the time of survey development and early administration, though this language later changed to “weekly ECHO sessions”. PASC was also used to describe what we later call Long COVID)).*

**Clinician 3M**

| **1. First Name*** | ______________________________ |
| --- | --- |
| **2. Last Name*** | ______________________________ |

**Clinician Knowledge**

| **3. My knowledge of appropriate PASC diagnosis has improved as a result of participation in the session.*** | ☐ Strongly Disagree ☐ Disagree ☐ Neutral ☐ Agree ☐ Strongly Agree |
| --- | --- |
| **4. My knowledge of effective PASC care and treatment has improved as a result of participation in the session.*** | ☐ Strongly Disagree ☐ Disagree ☐ Neutral ☐ Agree ☐ Strongly Agree |
| **5. My knowledge of approaches for complex cases of PASC has improved as a result of participation in these sessions.*** | ☐ Strongly Disagree ☐ Disagree ☐ Neutral ☐ Agree ☐ Strongly Agree |

**Clinician Confidence**

| **6. I am confident in my ability to identify tools and methods for PASC care.*** | ☐ Strongly Disagree ☐ Disagree ☐ Neutral ☐ Agree ☐ Strongly Agree |
| --- | --- |
| **7. I am confident in my ability to diagnose patients with PASC.*** | ☐ Strongly Disagree ☐ Disagree ☐ Neutral ☐ Agree ☐ Strongly Agree |
| **8. I am confident in my ability to screen for co-morbidities of PASC.*** | ☐ Strongly Disagree ☐ Disagree ☐ Neutral ☐ Agree ☐ Strongly Agree |
| **9. I am confident in my ability to generate a patient-specific treatment plan for patients with PASC.*** | ☐ Strongly Disagree ☐ Disagree ☐ Neutral ☐ Agree ☐ Strongly Agree |
| **10. I am confident in my ability to implement a patient-specific treatment plan for patients with PASC.*** | ☐ Strongly Disagree ☐ Disagree ☐ Neutral ☐ Agree ☐ Strongly Agree |
| **11. I am confident in my ability to select appropriate special or diagnostic tests for PASC patients.*** | ☐ Strongly Disagree ☐ Disagree ☐ Neutral ☐ Agree ☐ Strongly Agree |
| **12. I am confident in my ability to interpret special or diagnostic test results for my PASC patients.*** | ☐ Strongly Disagree ☐ Disagree ☐ Neutral ☐ Agree ☐ Strongly Agree |
| **13. I am confident in my ability to provide appropriate patient education about their PASC condition.*** | ☐ Strongly Disagree ☐ Disagree ☐ Neutral ☐ Agree ☐ Strongly Agree |
| **14. I am confident in my ability to educate clinic staff about PASC care.*** | ☐ Strongly Disagree ☐ Disagree ☐ Neutral ☐ Agree ☐ Strongly Agree |
| **15. I am confident in my ability to address PASC patients using a multidisciplinary approach.*** | ☐ Strongly Disagree ☐ Disagree ☐ Neutral ☐ Agree ☐ Strongly Agree |
| **16. I am confident in my ability to assess and manage concerns that PASC patients face.*** | ☐ Strongly Disagree ☐ Disagree ☐ Neutral ☐ Agree ☐ Strongly Agree |
| **17. I am confident in my ability to assess severity of disease in patients with PASC.*** | ☐ Strongly Disagree ☐ Disagree ☐ Neutral ☐ Agree ☐ Strongly Agree |
| **18. I am confident in my ability to deal with complex cases of PASC.*** | ☐ Strongly Disagree ☐ Disagree ☐ Neutral ☐ Agree ☐ Strongly Agree |

**Satisfaction**

| **19. Have you encountered a case similar to one presented during a previous ECHO session? *** | ☐ Yes ☐ No |
| --- | --- |
| **20. My overall satisfaction with the teleECHOs are:*** | ☐ Strongly Satisfied ☐ Satisfied ☐ Neutral ☐ Dissatisfied ☐ Strongly Dissatisfied |
| **21. Would you recommend this teleECHO colleague?*** | ☐ Definitely Not ☐ Probably Not ☐ Possibly ☐ Probably Yes ☐ Definitively Yes |
| **22. Would you participate in a future teleECHO like this one?*** | ☐ Definitely Not ☐ Probably Not ☐ Possibly ☐ Probably Yes ☐ Definitively Yes |

**Changes to Practice**

| **23. Have you helped a colleague care for their patient based on information learned from these weekly teleECHOs?*** | ☐ Yes ☐ No |
| --- | --- |
| **24. As a result of participating in the weekly teleECHO: I have a better understanding of further monitoring and follow-up plan.*** | ☐ Strongly Disagree ☐ Disagree ☐ Neutral ☐ Agree ☐ Strongly Agree |
| **25. As a result of participating in the weekly teleECHO: I developed more appropriate treatment plans.*** | ☐ Strongly Disagree ☐ Disagree ☐ Neutral ☐ Agree ☐ Strongly Agree |
| **26. As a result of participating in the weekly teleECHO session: I have a better understanding of underlying disease process.*** | ☐ Strongly Disagree ☐ Disagree ☐ Neutral ☐ Agree ☐ Strongly Agree |
| **27. As a result of participating in the weekly teleECHO session: I have a better understanding of the role of specialist referral.*** | ☐ Strongly Disagree ☐ Disagree ☐ Neutral ☐ Agree ☐ Strongly Agree |
| **28. As a result of participating in the weekly teleECHO session: I was able to more independently manage my PASC patients without referral to a specialist.*** | ☐ Strongly Disagree ☐ Disagree ☐ Neutral ☐ Agree ☐ Strongly Agree |
| **29. As a result of participating in the weekly teleECHO session: I was not able to apply what I learned. *** | ☐ Strongly Disagree ☐ Disagree ☐ Neutral ☐ Agree ☐ Strongly Agree |
| **30. As a result of participating in the weekly teleECHO session: I was able to apply specialist recommendations to patient care that I learned in this series.*** | ☐ Strongly Disagree ☐ Disagree ☐ Neutral ☐ Agree ☐ Strongly Agree |
| **31. As a result of participating in the weekly teleECHO session: I made other changes to my practice.*** | ______________________________________________________________ ______________________________________________________________ |

**Weekly ECHO Session Survey**

(*Publication note: This survey was administered to intervention clinicians that participated in the respective session. “Tele-ECHO” was used to describe the weekly sessions at the time of survey development and early administration, though this language later changed to “weekly ECHO sessions”.)*

| **1. Rate your knowledge of the topic before the session** | ☐ Not at all knowledgeable ☐ Slightly knowledgeable ☐ Moderately knowledgeable ☐ Very knowledgeable ☐ Extremely knowledgeable |
| --- | --- |
| **2. Rate your knowledge of the topic after the session** | ☐ Not at all knowledgeable ☐ Slightly knowledgeable ☐ Moderately knowledgeable ☐ Very knowledgeable ☐ Extremely knowledgeable |
| **3. How well were the stated objectives met?** | ☐ Excellent ☐ Very good ☐ Good ☐ Fair ☐ Poor |
| **4. How well did the session deliver balanced and objective, evidence-based content?*** | ☐ Excellent ☐ Very good ☐ Good ☐ Fair ☐ Poor |
| **5. The pace of the session was*** | ☐ Excellent ☐ Very good ☐ Good ☐ Fair ☐ Poor |
| **6. Opportunities to ask questions were*** | ☐ Excellent ☐ Very good ☐ Good ☐ Fair ☐ Poor |
| **7. Organization of the presenters' presentations were*** | ☐ Excellent ☐ Very good ☐ Good ☐ Fair ☐ Poor |
| **8. The presenters' ability to clearly communicate was*** | ☐ Excellent ☐ Very good ☐ Good ☐ Fair ☐ Poor |
| **9. What would you like to learn more about in future sessions?*** | __________________________________________________________________________________________ |
| **10. Was the source of evidence presented?*** | ☐ Yes ☐ No |
| **11. Was there commercial bias in this session?*** | ☐ Yes ☐ No |
| **12. If yes, please describe the commercial bias*** | _____________________________________________ _____________________________________________ |
| **13. Do you have any recommendations for how we may improve future sessions?** | _____________________________________________ _____________________________________________ |
| **14. How relevant was this session to your current work?** | ☐ Not at all relevant ☐ Slightly relevant ☐ Moderately relevant ☐ Very relevant ☐ Extremely relevant |
| **15. Will you use what you learned in this session in your work?** | ☐ Yes ☐ No If yes, please explain. _____________________________________________ _____________________________________________ |
| **16. What did you like best about this session?** | __________________________________________________________________________________________ |
| **17. What did you like least about this session?** | _____________________________________________ _____________________________________________ |
| **18. Would you recommend these sessions to a colleague?** | ☐ Definitely not ☐ Probably not ☐ Possibly ☐ Probably yes ☐ Definitely yes |
| **19. Changes that I am going to make in my practice:** | _____________________________________________ _____________________________________________ |
| **20. If no changes, what are the barriers** | __________________________________________________________________________________________ |

Note: Questions marked with an * were required by University of New Mexico to award continuing medical education credit for attending the session and were not used in the study.

**Monthly Webinar Survey**

(*Publication note: This survey was administered to control and intervention clinicians participating in the respective webinar*).

| **1. Rate your knowledge of the topic before the session** | ☐ Not at all knowledgeable ☐ Slightly knowledgeable ☐ Moderately knowledgeable ☐ Very knowledgeable ☐ Extremely knowledgeable |
| --- | --- |
| **2. Rate your knowledge of the topic after the session** | ☐ Not at all knowledgeable ☐ Slightly knowledgeable ☐ Moderately knowledgeable ☐ Very knowledgeable ☐ Extremely knowledgeable |
| **3. Will you use what you learned in this session in your work?** | ☐ Yes ☐ No If yes, please explain.  _____________________________________________ _____________________________________________ |
| **4. How well were the stated objectives met?** | ☐ Excellent ☐ Very good ☐ Good ☐ Fair ☐ Poor |
| **5. How well did the session deliver balanced and objective, evidence-based content?*** | ☐ Excellent ☐ Very good ☐ Good ☐ Fair ☐ Poor |
| **6. The pace of the session was*** | ☐ Excellent ☐ Very good ☐ Good ☐ Fair ☐ Poor |
| **7. Opportunities to ask questions were*** | ☐ Excellent ☐ Very good ☐ Good ☐ Fair ☐ Poor |
| **8. Organization of the presenters' presentations were*** | ☐ Excellent ☐ Very good ☐ Good ☐ Fair ☐ Poor |
| **9. The presenters' ability to clearly communicate was*** | ☐ Excellent ☐ Very good ☐ Good ☐ Fair ☐ Poor |
| **10. What would you like to learn more about in future sessions?** | _____________________________________________ _____________________________________________ |
| **11. Was the source of evidence presented?*** | ☐ Yes ☐ No |
| **12. Was there commercial bias in this session?*** | ☐ Yes ☐ No |
| **13. If yes, please describe the commercial bias*** | _____________________________________________ _____________________________________________ |
| **14. What did you like best about this session?** | _____________________________________________ _____________________________________________ |
| **15. What did you like least about this session?** | __________________________________________________________________________________________ |
| **16. Do you have any recommendations for how we may improve future sessions?** | _____________________________________________ _____________________________________________ |
| **17. How relevant was this session to your current work?** | ☐ Not at all relevant ☐ Slightly relevant ☐ Moderately relevant ☐ Very relevant ☐ Extremely relevant |
| **18. Changes that I am going to make in my practice:** | _____________________________________________ _____________________________________________ |
| **19. If no changes, what are the barriers** | _____________________________________________ _____________________________________________ |
| **20. Would you recommend these sessions to a colleague?** | ☐ Definitely not ☐ Probably not ☐ Possibly ☐ Probably yes ☐ Definitely yes |
| **21. I intend to apply the knowledge and/or skills I have acquired from this activity to my work when in a team environment.** | ☐ Strongly agree ☐ Agree ☐ Neutral ☐ Disagree ☐ Strongly Disagree |
| **22. I am better able to communicate with other members of a multidisciplinary team as a result of what I learned in this activity.** | ☐ Strongly agree ☐ Agree ☐ Neutral ☐ Disagree ☐ Strongly Disagree |
| **23. I am better able to discuss how teamwork can contribute to continuous and reliable patient care.** | ☐ Strongly agree ☐ Agree ☐ Neutral ☐ Disagree ☐ Strongly Disagree |

Note: Questions marked with an * were required by University of New Mexico to award continuing medical education credit for attending the session and were not used in the study.

**Quarterly Asynchronous Short Course Survey**

*(Publication note: This survey was administered to all trial clinicians that completed the respective quarterly asynchronous short course).*

| **1. Rate your knowledge of the topic before the course** | ☐ Not at all knowledgeable ☐ Slightly knowledgeable ☐ Moderately knowledgeable ☐ Very knowledgeable ☐ Extremely knowledgeable |
| --- | --- |
| **2. Rate your knowledge of the topic after the course** | ☐ Not at all knowledgeable ☐ Slightly knowledgeable ☐ Moderately knowledgeable ☐ Very knowledgeable ☐ Extremely knowledgeable |
| **3. Will you use what you learned in this course in your work?** | ☐ Yes ☐ No If yes, please explain.  ________________________________________________________________ ________________________________________________________________ |
| **4. How well were the stated objectives met?** | ☐ Excellent ☐ Very good ☐ Good ☐ Fair ☐ Poor |
| **5. What did you like best about this course?** | ________________________________________________________________ ________________________________________________________________ |
| **6. What did you like least about this course?** | ________________________________________________________________ ________________________________________________________________ |
| **7. How relevant was this course to your current work?** | ☐ Not at all relevant ☐ Slightly relevant ☐ Moderately relevant ☐ Very relevant ☐ Extremely relevant |
| **8. Changes that I am going to make in my practice:** | ________________________________________________________________ ________________________________________________________________ |
| **9. If no changes, what are the barriers** | ________________________________________________________________ ________________________________________________________________ |
| **10. Would you recommend these courses to a colleague?** | ☐ Definitely not ☐ Probably not ☐ Possibly ☐ Probably yes ☐ Definitely yes |

# **Qualitative Interview Guide: Clinical Provider, Intervention**

### *(Publication note: This interview was administered to intervention clinicians.)*

**Introduction:** My name is ____, a co-investigator of the U.S. CDC Long Covid & Fatiguing Illnesses Recovery Program, which is a three year long randomized control study. You were selected for this interview because of your participation in this study. Your participation was as a clinical provider of patients with long COVID & other fatiguing illnesses at Family Health Services of San Diego, in the intervention group. As a member of this study population, you attended weekly virtual extensions of community healthcare outcome **(ECHO)** sessions related to patient care for long COVID & other fatiguing illnesses, and may have participated in monthly webinars and/or quarterly short courses.

The purpose of this interview is to solicit your qualitative feedback on the **ECHO** series. I will ask you about what challenges you faced in participating and any benefits you perceived in participation. Lastly, I will ask you for any recommendations for improving the intervention. This interview should last about 30 minutes. Your participation in the interview is voluntary and can be stopped at any time. Your responses are anonymous and confidential. Our interview will be video recorded for the purposes of transcription, and will only viewed by myself and the transcriber. Do you have any questions for me before we begin?

**Your experience of the ECHO series**

My first questions focus on your personal perspective of the **ECHO** series quality and accessibility.

1. Did you face any **challenges** to participating in the weekly sessions? If so, what were they?
a. Would any changes to the **ECHO** series make it easier for you to participate?
b. Did you view video recordings of the sessions when you couldn’t attend? Were they easy to access?
2. Would you **attend ECHO sessions in the future** outside of this study, and if so, at what frequency (weekly, biweekly, monthly, quarterly)?

**Impacts of the series**

The next questions will ask you to explain any impacts- positive or negative- that resulted from your participation in the **ECHO** series.

3. What were **the benefits you perceived of participating** in the series to you personally or professionally?
4. What was the most **important impact** of the **ECHO** series on your care for patients with long covid and other fatiguing illnesses? Please describe any **changes to practice.**
5. Did the **quality of your care** for patients improve as a result of participating in the series? Please explain.

- *Interviewer probes what factors from the ECHO most influenced quality- i.e. didactics, case studies, engagement with specialists, offline conversations with specialists, etc.*

6. Did you experience an **improvement in the number of Long COVID patients you could manage** at any given time as a result of the series? (i.e. were you able to increase your patient panel?) If Yes, please explain. (Why or why not, how?)

- *Interviewer probes major drivers of this change- i.e. More efficient referrals? Less frequent patient visits? Greater overall demand? Or are they becoming known for their expertise on the subject in their clinic setting?*

7. Did the participation of **lived experience experts** influence any changes to your practice? Why or why not?
8. Did your participation in the **ECHO** series **influence other providers** in your clinic? Please explain. (Why or why not, how?)

- *Interviewer probes how interviewee shared information/ knowledge with other providers, which other providers, and in what settings, and whether this impacted their patient care.*

9. Did your **relationships with specialists** change or improve as a result of the **ECHO** series? Please explain. (why or why not, how?)

- *Interviewer probes what specialist relationships have improved, and how*
- *Did your understanding of specialties that could be referred to change or improve?*

**Improving the Series**

The last questions ask for feedback that we can use to inform to sustain and expand the series.

10. What do you see as the **greatest barriers** for the sustainability or scalability of this **ECHO** series in the future?
11. What **recommendations** do you have for those that wish to sustain and scale this **ECHO** series for clinicians providing care to long Covid & other fatiguing illness patients?

This is the end of our interview. We will transcribe these interviews anonymously and conduct analysis for publication, and for recommendations regarding the future of this **ECHO** series. Do you have any questions for me? Or other comments or information you’d like me to know?

# **Qualitative Interview Guide: Control Group Clinicians**

*(Publication note: This interview was administered to control clinicians.)*

**Introduction:** My name is ____, a co-investigator of the U.S. CDC Long Covid & Fatiguing Illnesses Recovery Program, which is a three year long randomized control study. You were selected for this interview because of your participation in this study. Your participation was as a clinical provider of patients with long COVID & other fatiguing illnesses at Family Health Services of San Diego, in the control group. As a member of this study population, you attended monthly webinars and/or quarterly short courses.

The purpose of this interview is to solicit your qualitative feedback on these educational offerings. I will ask you about what challenges you faced in participating and any benefits you perceived in participation. Lastly, I will ask you for any recommendations for improving the interventions. This interview should last about 30 minutes. Your participation in the interview is voluntary and can be stopped at any time. Your responses are anonymous and confidential. Our interview will be video recorded for the purposes of transcription, and will only viewed by myself and the transcriber. Do you have any questions for me before we begin?

**Your experience of the ECHO series**

My first questions focus on your personal perspective of the quality and accessibility of the monthly webinars and quarterly short courses.

1. Did you face any **challenges to participating** in the monthly webinars or quarterly short courses? If so, what were they?
a. Would any changes to these offerings make it easier for you to participate?
2. Would you **attend these offerings in the future** outside of this study, and if so, at what frequency (less frequent, about the same, more frequent than offered in the study)

**Impacts of the series**

The next questions will ask you to explain any impacts- positive or negative- that resulted from your participation in the monthly webinars and quarterly short courses.

3. What benefits did you perceive to participating in the series?
4. What was the most **important impact** of the monthly webinars and quarterly short courses on your care for patients with long covid and other fatiguing illnesses? Please describe any **changes to practice**.
5. Did the **quality of your care** for patients improve as a result of participating in the monthly webinars or quarterly short courses? Please explain. (Why or why not, how?)

- *Interviewer probes what factors from offerings most influenced quality- i.e. didactics, opportunities to ask questions of presenters, etc.*

6. Did the participation of **lived experience experts** influence any changes to your practice? Why or why not?
7. Did you perceive any differences in this impact between the monthly webinar or the quarterly short course?
8. Did you experience an **improvement in the number of Long COVID patients** you could manage at any given time as a result of these offerings? (i.e. were you able to increase your patient panel?) If Yes, please explain. (Why or why not, how?)

- *Interviewer probes major drivers of this change- i.e. More efficient referrals? Less frequent patient visits? Greater overall demand? Or are they becoming known for their expertise on the subject in their clinic setting?*

9. Did your participation in the monthly webinars or quarterly short courses **influence other providers** in your clinic? Please explain. (Why or why not, how?)

- *Interviewer probes how interviewee shared information/ knowledge with other providers, which other providers, and in what settings, and whether this impacted their patient care.*

10. Did your **relationships with specialists** change or improve as a result of the **these offerings**? Please explain. (why or why not, how?)

- *Interviewer probes what specialist relationships have improved, and how*
- *Did your knowledge of specialities that could be referred to change or improve?*

11. Did you perceive any **changes in your ability to manage patients independently vs. referring to specialists** as a result of these offerings?
12. Regarding all the benefits and impacts of the series you’ve mentioned, do you feel they were influenced more by the **monthly webinar or the quarterly short course**? Or where they influenced the same? I.e. was one more impactful than another?

**Improving the Series**

The last questions seek feedback that we can use to inform to sustain and expand the series.

13. What do you see as the **greatest barriers** for the sustainability or scalability of offering the monthly webinars and short courses in the future?
14. What **recommendations** do you have for those that wish to sustain and scale these educational offerings for clinicians providing care to long Covid & other fatiguing illness patients?

This is the end of our interview. We will transcribe these interviews and conduct analysis for publication, and for recommendations regarding the future of this series. Do you have any questions for me? Or other comments or information you’d like me to know?

# **Qualitative Interview Guide: Clinic Administrators**

*(Publication note: This interview was administered to clinic administrators.)*

**Introduction:** My name is ____, a co-investigator of the U.S. CDC Long Covid & Fatiguing Illnesses Recovery Program, which is a three year long randomized control study implemented by the Family Health Services of San Diego. You were selected for this interview because clinicians in your clinic participated in this study. The clinicians in your clinic were part of <intervention/ control> group of the study. As members of the <intervention/ control> group, your clinicians <attended weekly virtual extensions of community healthcare outcome **(ECHO)** sessions related to patient care for long COVID & other fatiguing illnesses and/or may have participated in monthly webinars and/or quarterly short courses>.

The purpose of this interview is to solicit your qualitative feedback on the impact of the trial’s interventions on the delivery of care for people living with Long Covid and fatiguing illnesses at your clinic, such as Myalgic encephalomyelitis/chronic fatigue syndrome (ME/CFS). I will probe whether you think the interventions improved the quality or efficiency of care delivery and what, if any, drawbacks exist from participating in the model. Lastly, I will ask you for any recommendations for improving the scalability and sustainability of the model. This interview should last about 30 minutes. Your participation in the interview is voluntary and can be stopped at any time. Your responses are anonymous and confidential and will only be viewed by our study team. Do you have any questions for me before we begin?

**Setting the Scene**

1. Do you have knowledge of which clinicians in your clinic participated in the trial, and their study status (control/ intervention)?

**Benefits and Drawbacks of Participation**

2. What benefits to the clinic- if any- did you observe as a result of the participation of your clinicians in study? Did you see any differential benefits to any specific educational offering?
3. What drawbacks to the clinic- if any- did you observe as a result of the participation of your clinicians in the study? Did you see any differences in the drawbacks experienced based on the educational offerings they attended?

**Impact on Care**

4. Did you observe any **increase in the panel composition of patients** living with Long COVID that you attribute to your clinician participation in the study? If so, please explain.
a. For example, did you perceive an improvement in the number of patients your participating clinicians could manage as a result of enhanced knowledge and skills gained from participating in the study?
5. Did the **quality** of care for patients with Long COVID and/or other fatiguing illnesses improve as a result of the study? If so, please explain.
6. Did the **efficiency** of care delivery improve for patients with Long COVID and/or other fatiguing illnesses as a result of the study? If so, please explain. For example, did you observe any changes to referral practices as a result of participation in the study?
7. Did you observe participating providers **sharing skills or knowledge gained** with non-participating clinicians at any point? If so, please elaborate.
8. What recommendations do you have for **improving the scalability or sustainability** of the model in the future?
9. What do you think would **motivate other clinical administrators to support their providers** to participate in a series like this one?

This is the end of our interview. We will transcribe these interviews anonymously and conduct analysis for publication, and for recommendations regarding the future of this series. Do you have any questions for me? Or other comments or information you’d like me to know?

# **Qualitative Interview Guide: Multi-disciplinary Specialists**

*(Publication note: This interview was administered to subject matter experts.)*

**Introduction:** My name is ____, a co-investigator of the U.S. CDC Long Covid & Fatiguing Illnesses Recovery Program, which is a three year long randomized control study implemented by the Family Health Services of San Diego. You were selected for this interview because you participated in this study as a specialist. As a member of the multi-disciplinary specialist group, you offered advice on patient care for long COVID & other fatiguing illnesses in the weekly **ECHO** sessions.

The purpose of this interview is to solicit your qualitative feedback on the impact of the trial’s interventions on the delivery of care for people living with Long Covid and other fatiguing illnesses at your clinic. I will probe any benefits or challenges you experienced participating in the study, and any impacts you observed to providers or patients. Lastly, I will ask you for any recommendations for improving the scalability and sustainability of the model. This interview should last about 30 minutes. Your participation in the interview is voluntary and can be stopped at any time. Your responses are anonymous and confidential. Our interview will be videorecorded for the purposes of transcription, and will only viewed by myself and the transcriber. Do you have any questions for me before we begin?

1. Did you face any challenges to participating in the weekly sessions? If so, what were they?
a. Would any changes to the **ECHO** series make it easier for you to participate?
2. What benefits did you experience personally from participating in the series?

Probe: Use of What’s app group and benefits.

a. Did you use the What’s app group? If so, did you obtain any benefits from it?
b. Did you find the video recordings and session materials access from previous sessions?
3. What benefits did you observe to the learning clinicians (from San Diego) participating in the **ECHO** series?
4. Did you observe a change in the complexity or type of cases presented, or questions posed, by providers throughout the duration of the study? Please explain.
5. Did your relationships with providers and/or specialists change or improve, as a result of the **ECHO** series? Please explain. (What relationships, how?)
6. Would you participate as a specialist in an **ECHO** series like this one again in the future? If so, at what frequency (weekly, monthly, quarterly)?
7. What do you see as the greatest barriers for the sustainability or scalability of this **ECHO** series in the future?
8. What recommendations do you have for those that wish to sustain and scale this **ECHO** series for clinicians providing care to long Covid & other fatiguing illness patients?

This is the end of our interview. We will transcribe these interviews anonymously and conduct analysis for publication, and for recommendations regarding the future of this **ECHO** series. Do you have any questions for me? Or other comments or information you’d like me to know?

**LC&FIRP Educational Session Topics**

# **1. Weekly ECHO Session Topics**

| **Session Number** | **Topic** |
| --- | --- |
| 1 | Intro to LC&FIRP |
| 2 | Diagnostic Criteria PASC, Definition of ME/CFS |
| 3 | Overview of Manifestations of PASC, Pulmonary Manifestations |
| 4 | General approach to management in Primary Care |
| 5 | Overview of ME/CFS |
| 6 | Role of Community Support |
| 7 | Research Update |
| 8 | Autonomic Testing and Treatment |
| 9 | Neurocognitive Function & Emotional Well-Being Post-Covid |
| 10 | Behavioral Interventions for Symptoms Management |
| 11 | Approach to Breathing Discomfort |
| 12 | The “4-P” model of Fatigue: Pacing, Prioritizing, Planning, Positioning |
| 13 | Ethical considerations in long-term treatment of conditions |
| 14 | Post-COVID Condition Related Fatigue |
| 15 | Mast cell activation syndrome (MCAS) |
| 16 | Self-management of autonomic issues |
| 17 | Research Update - Healthcare Disparities |
| 18 | Proposed Mechanisms for Cognitive Impairment |
| 19 | Post COVID Dysautonomia; diagnosis and initial management, a practical approach |
| 20 | Physical Therapy Interventions for PEM |
| 21 | Patient Support and Resources |
| 22 | Therapeutics |
| 23 | Diagnosing POTS |
| 24 | Cognitive Therapy Techniques for Post-COVID Conditions |
| 25 | Vestibular Symptom Management for Post-COVID Conditions |
| 26 | Research Update |
| 27 | Comorbidity factors affecting Post-COVID Conditions |
| 28 | Disability in CA for Post-COVID Conditions |
| 29 | Sleep and Post-COVID Conditions |
| 30 | Neuromuscular Issues |
| 31 | Mood and Post-COVID Conditions |
| 32 | Post-COVID Diabetes |
| 33 | Provider Self-Care |
| 34 | Lifestyle Changes Beyond Treatment |
| 35 | Journal Club |
| 36 | Integrative Medicine Approaches for Post-COVID Conditions |
| 37 | Changing Unhealthy Behaviors |
| 38 | Long COVID PACT Guide |
| 39 | Long-Term Cardiovascular Complications of COVID-19 |
| 40 | PTSD and Post-COVID Conditions |
| 41 | How to structure your first visit |
| 42 | Social Work Interventions |
| 43 | Nutrition for Post-COVID Conditions |
| 44 | Journal Club - https://pubmed.ncbi.nlm.nih.gov/36341457/ |
| 45 | Journal Club - Nutrition Update |
| 46 | Post-COVID Tinnitus |
| 47 | Evidence Review on LDN |
| 48 | Evidence Review on Stimulants |
| 49 | Journal Club - https://www.nature.com/articles/s41579-022-00846-2 |
| 50 | Sexual Dysfunction and Dysautonomia |
| 51 | Medical Trauma |
| 52 | Research Update - Cognitive Rehab |
| 53 | Gastrointestinal Manifestations of Post-COVID Conditions |
| 54 | Research Update - Rehabilitation |
| 55 | Dysautonomia and Post-COVID Conditions |
| 56 | Journal Club - Metformin and Long COVID https://www.bmj.com/content/381/bmj.p1306 |
| 57 | Research Update - PCC and Mental Health Comorbidities |
| 58 | Research Update - Fatigue |
| 59 | Research Update - https://pubmed.ncbi.nlm.nih.gov/35255491/ |
| 60 | Journal Club - https://onlinelibrary.wiley.com/doi/epdf/10.1002/pmrj.12894 |
| 61 | Evidence Review on Alternative Therapies |
| 62 | OT - Sensory Overload |
| 63 | Risk of MI and Stroke after COVID-19 |
| 64 | Patient Resource Update |
| 65 | Evidence Review Pyridostigmine |
| 66 | Journal Club - https://www.sciencedirect.com/science/article/abs/pii/S0889159123002374 |
| 67 | Journal Club - Developing a Definition of PASC https://jamanetwork.com/journals/jama/fullarticle/2805540 |
| 68 | Cervical Cranial Instability (CCI) |
| 69 | Evidence Review Cromolyn, Guanfacine, and Hyperbaric oxygen |
| 70 | Neuroinflammation, Brain fog, and the kynurenine pathway in Long COVID |
| 71 | Journal Club - https://www.nature.com/articles/s41467-023-44432-3 |
| 72 | Ehlers Danlos Syndrome |
| 73 | Journal Club - https://doi.org/10.1016/j.cell.2023.09.013 |
| 74 | POTS Treatment |
| 75 | Evidence Review NADPH |
| 76 | Journal Club - https://www.nature.com/articles/s41467-024-45107-3 |
| 77 | Journal Club - https://www.thelancet.com/journals/laninf/article/PIIS1473-3099(23)00684-9/fulltext |
| 78 | Journal Club - https://www.nature.com/articles/s41593-024-01576-9 |
| 79 | COVID triggering neurodegeneration |
| 80 | Policy Meeting Recap and Open Discussion |
| 81 | Journal Club - https://www.medrxiv.org/content/10.1101/2024.02.29.24303568v1.full.pdf |
| 82 | POTS Medication - Case study |
| 83 | IVIG |
| 84 | Long COVID and POTS Research Trials |
| 85 | Baseline Data Review of Alternative Therapies |
| 86 | NASEM Long COVID definition https://nap.nationalacademies.org/read/27768/chapter/1 |
| 87 | Patient Perspective on recreational therapies for LC&FI |
| 88 | RECOVER Neuro |
| 89 | Journal Club Pediatrics - https://jamanetwork.com/journals/jama/article-abstract/2822770 |

# **2. Monthly Webinar Topics**

| **Session Number** | **Topic** |
| --- | --- |
| 1 | Overview of PASC, ME/CFS, OPIFI |
| 2 | Neurocognitive Manifestations of Post-COVID Conditions |
| 3 | Pulmonary Manifestations of Post-COVID Conditions |
| 4 | Autonomic/Cardiac Manifestations of Post-COVID Conditions |
| 5 | History of ME/CFS |
| 6 | The Patient Perspective on Post-Infectious Fatiguing Illnesses |
| 7 | Practical Strategies for Symptom Management |
| 8 | Physical Therapy and Post-COVID Conditions |
| 9 | Pediatric Post-COVID Conditions |
| 10 | Research Update |
| 11 | Disability and Post-COVID Conditions |
| 12 | Occupational Medicine and Post-COVID Conditions |
| 13 | Back to Basics |
| 14 | Occupational and Speech Therapy for Post-COVID Conditions |
| 15 | Mood and Post-COVID Conditions |
| 16 | Research Update on Microclots (*and their role on the pathiophysiology of Long COVID) |
| 17 | Mast Cell Activation Syndrome (MCAS) |
| 18 | Low-Dose Naltrexone (LDN) |
| 19 | GI Issues and Nutrition for Post-COVID Conditions |
| 20 | Patient led research in Post-COVID Conditions |
| 21 | Integrative Medicine Post-COVID Conditions Group Approach |
| 22 | Research Update - RECOVER |
| 23 | Cardiovascular Outcomes in Post-COVID Conditions |
| 24 | Journal Club |
| 25 | Long COVID Symptomatic Treatment Update |
| 26 | Sleep and Post-COVID Conditions |
| 27 | Patient Resources and Support |
| 28 | Low-dose naltrexone use for the management of post-acute sequelae of COVID-19 |
| 29 | PEM Diagnosis |
| 30 | Repurposed Therapies and COVID OUT results /CURE ID |
| 31 | Impact of vaccination on Long COVID |
| 32 | Long COVID Patient Navigation |
| 33 | Stanford Paxlovid Results - https://jamanetwork.com/journals/jamainternalmedicine/fullarticle/2819901 / RECOVER AUTO Overview |
| 34 | Journal Club - https://academic.oup.com/ofid/article/11/9/ofae462/7733485 |

# **3. Quarterly Asynchronous Short Courses**

| **Course** | **Title and Modules** |
| --- | --- |
| 1 | Quarterly Course 1 for LC&FIRP  • Overview of Post-COVID Conditions  • Overlap with Post-Viral Syndromes and ME/CFS  • General Approach to Post-COVID Conditions in Primary Care  • Pulmonary, Autonomic, and Neurocognitive Manifestations |
| 2 | Clinical Approaches to Managing Post-COVID Conditions  • Introduction to Post-COVID Conditions “Long COVID”  • Management of Fatigue & Post-Exertional Malaise  • Management of Pulmonary & Autonomic Symptoms  • Patient & Ethical Considerations in Managing Chronic Illness  • Updates in Proposed Etiologies for Post-COVID Conditions |
| 3 | Quarterly Course 3 for LC&FIRP  • Clinical Presentation and Pathophysiology  • Diagnosis of POTS, Vestibular Symptoms, PEM and Brain Fog  • Self-Management of POTS, Vestibular Symptoms, PEM, Fatigue and Brain Fog  • Clinical Management |
| 4 | Quarterly Course 4 for LC&FIRP  • Updated Discussion of Post-COVID Conditions  • Post-COVID Diabetes and Other Comorbidities Associated with Post-COVID Conditions  • Mood and Sleep Symptoms Associated with Post-COVID Conditions  • Provider Self-care and Post-COVID Conditions Disability Resources |
| 5 | Brain Fog and Cognitive Impairment in Post-COVID Conditions  • Etiology and Epidemiology  • Risk Factors and Pathophysiology  • Clinical Management  • Self-Management and Patient Resources |
| 6 | Fatigue and Post-Exertional Malaise  • Definitions and Epidemiology  • Risk Factors and Pathophysiology  • Clinical Diagnosis  • Management |
| 7 | Post-infectious Disease Syndromes: A Precursor to Long COVID  • Overview of Post-infectious Disease Syndromes  • Proposed Etiologies of Post-Infectious Disease Syndromes  • Mechanism-specific Symptoms of Post-infectious Disease Syndromes |
| 8 | Long COVID Patient Experiences and Systems of Care  • Lessons from Patient Experiences with Long COVID  • Federal Initiatives to Improve Care in Long COVID  • Resources for Providers, Nurses and Patients |
| 9 | A Multidisciplinary Approach to Long COVID Care: Three Case Studies  • Overview of Long COVID  • Case Study: Autonomic Dysfunction in Long COVID  • Case Study: Post Exertional Malaise and Fatigue  • Brain Fog |
